# Stimulation of interferon-β responses by aberrant SARS-CoV-2 small viral RNAs acting as retinoic acid-inducible gene-I agonists

**DOI:** 10.1016/j.isci.2022.105742

**Published:** 2022-12-07

**Authors:** Yasuha Arai, Itaru Yamanaka, Toru Okamoto, Ayana Isobe, Naomi Nakai, Naoko Kamimura, Tatsuya Suzuki, Tomo Daidoji, Takao Ono, Takaaki Nakaya, Kazuhiko Matsumoto, Daisuke Okuzaki, Yohei Watanabe

**Affiliations:** 1 Department of Infectious Diseases, Kyoto Prefectural University of Medicine, Kyoto 602-8566, Japan; 2 Genome Information Research Center, Research Institute for Microbial Diseases, Osaka University, Osaka 565-0871, Japan; 3 Institute for Advanced Co-Creation Studies, Research Institute for Microbial Diseases, Osaka University, Osaka 565-0871, Japan; 4 SANKEN, Osaka University, Osaka 567-0047, Japan; 5 Single Cell Genomics, Human Immunology, WPI Immunology Frontier Research Center, Osaka University, Osaka 565-0871, Japan

**Keywords:** immunology and virology

## Abstract

Patients with severe COVID-19 exhibit a cytokine storm characterized by greatly elevated levels of cytokines. Despite this, the interferon (IFN) response is delayed, contributing to disease progression. Here, we report that SARS-CoV-2 excessively generates small viral RNAs (svRNAs) encoding exact 5′ ends of positive-sense genes in human cells *in vitro* and *ex vivo*, whereas endemic human coronaviruses (OC43 and 229E) produce significantly fewer similar svRNAs. SARS-CoV-2 5′ end svRNAs are RIG-I agonists and induce the IFN-β response in the later stages of infection. The first 60-nt ends bearing duplex structures and 5′-triphosphates are responsible for immune-stimulation. We propose that RIG-I activation by accumulated SARS-CoV-2 5′ end svRNAs may contribute to later drive over-exuberant IFN production. Additionally, the differences in the amounts of svRNAs produced and the corresponding IFN response among CoV strains suggest that lower svRNA production during replication may correlate with the weaker immune response seen in less pathogenic CoVs.

## Highlights

• SARS-CoV-2 overproduces small viral RNAs encoding exact 5′ end genome in human cells
• The 5′ end small viral RNAs are RIG-I stimulatory and act as IFN/cytokine agonists
• The 5′ end svRNA accumulates in cells *in vitro* and *ex vivo* at late infection stages
• svRNA accumulation in cells may confer delayed IFN response as in severe COVID-19

## Graphical abstract

**Figure:**
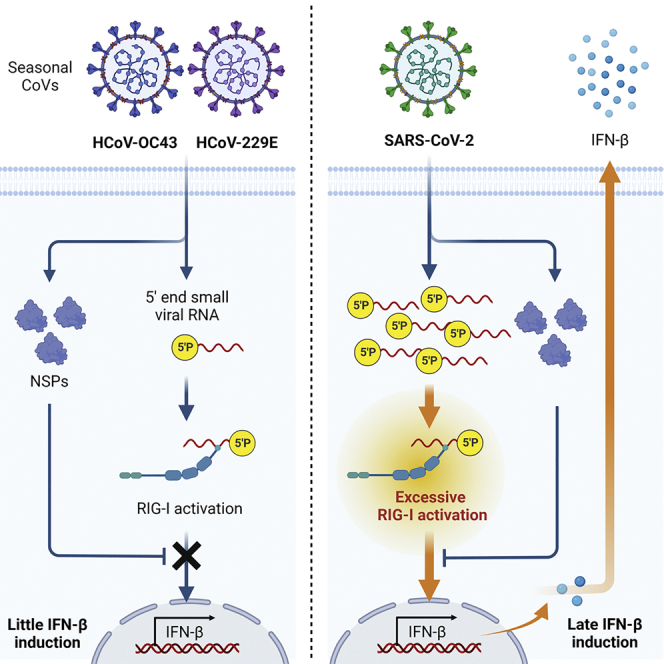

## Introduction

The coronavirus disease 2019 (COVID-19) pandemic, caused by severe acute respiratory syndrome coronavirus 2 (SARS-CoV-2), is an ongoing global health threat. Although the Delta variant (B.1.617.2) dominated previous variants and spread to >180 countries by September 2021,^1^ the newly emerging Omicron variants (B.1.1.529.1/BA.1 and B.1.1.529.2/BA.2) are outcompeting Delta and quickly becoming the dominant lineage globally as of February 2022.^2^ Patients with severe COVID-19 exhibit a hyperinflammatory response referred to as a “cytokine storm,”^3^ which is characterized by excessive levels of cytokines including interleukin (IL)-6 and a variety of different interferons (IFNs).^4^^,^^5^^,^^6^^,^^7^^,^^8^

Like most viral RNAs, coronavirus RNA is detected by host RNA sensors, cytosolic retinoic acid-inducible gene-I (RIG-I)-like receptors including RIG-I and melanoma differentiation-associated protein 5 (MDA-5).^9^^,^^10^^,^^11^ Upon activation, RIG-I and MDA-5 transduce signaling cascades, leading to the activation of interferon regulatory factor 3 (IRF3) and NF-κB that are required for type 1 IFN and pro-inflammatory cytokine production, respectively. During SARS-CoV-2 infection, both RIG-I and MDA5 reportedly sense SARS-CoV-2 RNAs to activate innate immune responses,^11^^,^^12^ although studies suggest that it is MDA5 that predominantly governs the innate immune response to SARS-CoV-2.^13^^,^^14^ However, it is not well understood which viral RNA species are sensed by these molecules on infection. Additionally, longitudinal analyses revealed that SARS-CoV-2 does elicit an IFN response, but this is delayed,^15^ suspected to contribute to disease progression.^16^^,^^17^^,^^18^ However, the viral mechanism driving the characteristic signature of delayed IFN induction in COVID-19 is poorly understood.

Abortive RNA production has been understood to be a result of RNA transcription by RNA polymerase of host machinery (e.g. abortive initiation by bacterial and eukaryotic RNA polymerase),^19^^,^^20^ host machinery during viral infection (e.g. abortive HIV-1 RNA transcripts consisting of TAR hairpin RNA)^21^^,^^22^ and RNA viruses (double-stranded RNA viruses and single-stranded RNA viruses with both polarities).^23^^,^^24^^,^^25^^,^^26^^,^^27^ It remains unclear how host and viral machinery are involved in the production of the abortive RNA transcripts. In particular, RNA viruses are known to exhibit high replication error rates during their reproduction and thereby produce abortive leader RNAs. It was suggested that these are recognized by RIG-I, thereby inducing an innate immune response.^27^^,^^28^^,^^29^ The emergent RNA virus SARS-CoV-2 is now in a transitional period moving from bats to human hosts^30^ and supposedly faces challenges in hijacking the replication machinery in human cells.^31^^,^^32^ Thus, SARS-CoV-2 replication in humans may produce erroneous RNAs in large amounts, which may trigger dysregulated host cell transcriptomic and immune responses, including the cytokine storm as seen with other highly pathogenic viruses such as some avian influenza virus strains.^27^ Although other human coronaviruses (HCoVs) also originated from non-human CoVs, they have already established stable virus lineages in humans over long adaptation periods. These considerations motivated us to hypothesize that the distinct production of aberrant viral RNAs among different CoVs may represent one factor responsible for the different outcomes in terms of immune responses and disease severity. Interplay between aberrant viral RNAs and COVID-19 pathogenesis in this context remains to be elucidated. Here, we applied deep sequencing, focusing on the small RNAs (<200-nt) produced by SARS-CoV-2 in comparison with two endemic HCoVs (229E and OC43) that cause common colds and more mildly symptomatic respiratory disease. We report that SARS-CoV-2 infection results in the generation of excessive amounts of small RNAs that act as RIG-I ligands to induce the IFN-β responses in later stages of infection, which may be associated with the pathology observed in severe COVID-19.

## Results

### SARS-CoV-2 replicates efficiently in cultured cells at levels similar to human coronaviruses

**Figure 1:**
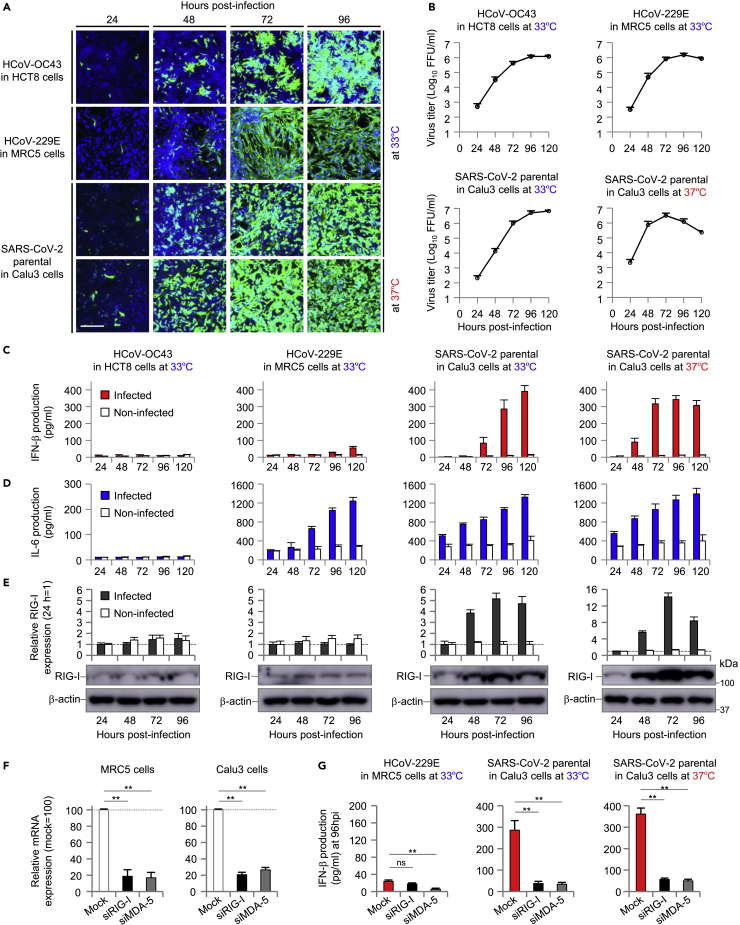
SARS-CoV2 stimulates IFN expression in the late stages of infection (A and B) Replication kinetics of SARS-CoV-2, HCoV-229E, and HCoV-OC43 in human cells. Cells were infected with CoVs at an MOI of 0.001 and incubated at 33°C (for HCoV-OC43 and HCoV-229E) and both at 33°C and 37°C (for SARS-CoV-2). (A) Confocal microscopy images of CoV-infected human cells. At the indicated times post-infection, cells were stained with antiviral NP antibodies (green). Nuclei were stained with Hoechst 33,342 (blue). Scale bar = 250 μm. (B) Progeny virus titers were measured by focus-forming assays at the indicated times post-infection. (C and D) IFN-β (C) and IL-6 (D) released from CoV-infected human cells. At the indicated times post-infection, the supernatants were harvested for ELISA. (E) Immunoblot analysis of RIG-I in lysates from CoV-infected human cells at the indicated times post-infection. Representative images from three independent experiments are shown. Quantification of band intensity is expressed relative to 24 h post-infection. β-actin levels are shown as loading controls. (F) RNAi-mediated depletion of RIG-I and MDA-5. The expression levels of RIG-I and MDA-5 for mock were set to 100%. (G) IFN-β released from siRNA-transfected cells. At 72 h (for MRC5 cells and Calu-3 cells at 33°C) or 48 h (for Calu-3 cells at 37°C) post-infection, the supernatants were harvested for ELISA. Each data point is the mean ± SD from three independent experiments. Statistically significant differences compared to mock transfected cells are determined by ANOVA with Tukey’s multiple comparison test and shown as ∗∗p < 0.01 (ns = not significant).

We initially determined the kinetics of replication of HCoV-OC43, HCoV-229E, and SARS-CoV-2 parental strain (hereafter referred to as SARS-CoV-2) in different cell lines. This was necessary because there is no single cell line available that is permissive to all three CoVs. We, therefore, used human cell lines (HCT8, MRC5, and Calu-3 cells) and infected HCT8 cells with HCoV-OC43, MRC5 cells with HCoV-229E and Calu-3 cells with SARS-CoV-2 at a MOI of 0.001. The CoVs are generally propagated at different temperatures, namely 33°C for HCoV-OC43 and HCoV-229E and 37°C for SARS-CoV-2. We, therefore, monitored the replication kinetics at 33°C for HCoV-OC43 and HCoV-229E and at both 33°C and 37°C for SARS-CoV-2 to exclude any potential bias due to different culture temperatures. All CoVs efficiently produced viral progeny, reaching almost 100% infection rates in the host cell populations and plateaued viral titers at the corresponding time-points at either temperature (Figures 1A and 1B). We also tested non-human primate Vero cells in this study, which are permissive to HCoV-OC43 and SARS-CoV-2 infections, and monitored in the same manner. The two CoVs also achieved 100% infection and plateaued viral titers at the corresponding time-points in Vero cells (Figures S1A and S1B). These data show that all CoVs completed a full replication cycle in these cells at either tested temperature and efficiently promulgated infection throughout the whole cell population with slightly different kinetics. For subsequent analyses, based on these results, CoV infections were performed at 33°C under the same conditions as above. RNAs were purified from the cells shortly before viral titers plateaued, at which time cytopathic effects were yet not apparent (in human cells 96 hpi for HCoVs and 72 hpi for SARS-CoV-2, and in Vero cells 96 hpi for HCoV-OC43 and 48 hpi for SARS-CoV-2) and CoV progeny virus titers were essentially similar.

### SARS-CoV-2 infection elicits an interferon response in human cells at a late stage of infection

Poly (I:C) robustly stimulated IFN-β and IL-6 secretion from HCT8, MRC5, and Calu-3 cells at 24 h post-transfection (Figures S2A and S2B), which correlated with the rapid induction of RIG-I expression (Figure S2C). These data confirm the presence of substantial intact antiviral signaling machinery in these established human cell lines.

We then infected these human cells with CoVs and measured the kinetics of IFN-β and IL-6 expression post-infection. The different CoVs evoked distinct antiviral responses in these cells (Figures 1C and 1D). HCoV-OC43 induced little IFN-β and IL-6 production over the whole time course, whereas HCoV-229E suppressed IFN-β production throughout the course of infection but IL-6 expression gradually increased over time. Notably, however, SARS-CoV-2 evoked both IFN-β and IL-6 expression at a later stage of infection regardless of culture temperature and correlated with the induction of RIG-I which was observed only in SARS-CoV-2-infected cells (Figure 1E). Depletion of cytoplasmic RNA sensors (RIG-I and MDA-5) reduced the inflammatory response after infection, suggesting that RNA sensing is a key driver of SARS-CoV-2-induced innate immune activation (Figures 1F and 1G). These data prompted us to identify the IFN-β agonistic RNAs active in SARS-CoV-2 infection. To this end, we focused on viral RNA species produced during SARS-CoV-2 replication in infected cells.

### SARS-CoV-2-derived small viral RNA species include interferon stimuli

**Figure 2:**
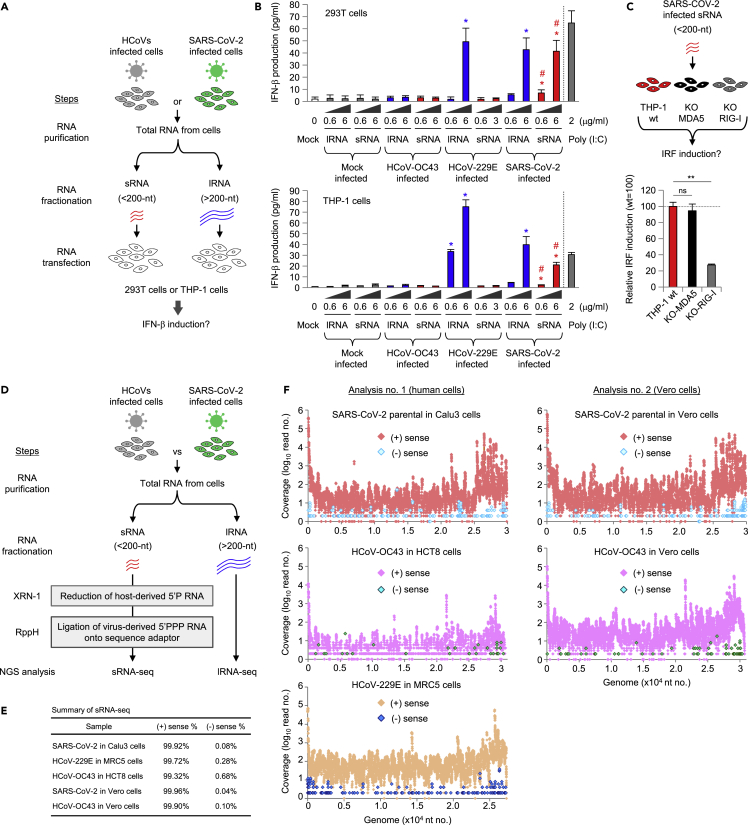
Small RNA fraction from SARS-CoV-2-infected cells, which mostly map to positive-strand genomes, has an immune-stimulatory capability in human cells (A) Schematic of RNA fractionation and transfection. (B) Induction of IFN-β and IL-6 secretion from 293T cells and differentiated THP-1 cells by the transfection of RNA fractions (0.6 μg/mL or 6 μg/mL) from Vero cells mock-infected or infected with CoVs. At 24 h after stimulation, the supernatants were harvested for ELISA. Supernatants from cells stimulated with poly (I:C) at 2 μg/mL were included as positive controls. Each data point is the mean ± SD from three independent experiments. Statistically significant differences compared to the cells stimulated with the same amount of RNA counterpart purified from mock- and HCoV-OC43-infected cells (∗p < 0.01) and from HCoV-229E-infected cells (#p < 0.01) are determined by ANOVA with Tukey’s multiple comparison test. (C) Induction of IRF/type 1 IFN signaling in THP-1 cells, THP-1-KO-MDA5 cells and THP-1-KO-RIG-I cells by the transfection of the sRNA fraction (2 μg/mL) from SARS-CoV-2-infected cells. (D) Schematic of RNA preparation for RNA-seq in this study. The RNA preparation method was previously established by others.^27^ (E) Summary of RNA reads mapped to CoV genomes. (F) sRNA-seq reads mapped to the viral genomes of CoVs. Reads were strand-specifically mapped to positive-sense (+) RNAs or negative-sense (−) RNAs. Read counts were quantified for each nucleotide of the genome.

We purified total RNAs from human cells mock-infected or infected with the different CoVs. The RNAs were separated into a large RNA (lRNA) fraction (>200-nt) and a small RNA (sRNA) fraction (<200-nt), which were then transfected into human 293T cells or differentiated THP-1 cells to assess their IFN-β stimulatory ability (Figure 2A). Neither RNA fraction from mock- or HCoV-OC43-infected cells stimulated IFN-β production above basal levels in the transfected cells (Figure 2B). The sRNA fractions from HCoV-229E-infected cells also failed to increase IFN-β levels, although the lRNA fraction raised them substantially above baseline. In contrast, both RNA fractions from SARS-CoV-2-infected cells stimulated IFN-β production to a similar degree as Poly (I:C). In addition, RIG-I-knockout THP-1 cells exhibited diminished IRF induction that leads to IFN production upon the activation of the sRNA fraction from SARS-CoV-2-infected cells, whereas MDA5-knockout THP-1 cells were similar to THP-1 wt cells in this respect (Figure 2C). This indicates that SARS-CoV-2 sRNA drives the RIG-I mediated IRF/Type 1 IFN pathway. These data show that sRNAs produced by SARS-CoV-2 are IFN-β stimulatory along with co-produced lRNAs. Because the IFN-β stimulatory activity of the small viral RNA (svRNA) fraction was unique to SARS-CoV-2 and likely to be associated with the increased IFN-β response during infection, as are also the longer viral RNAs, we then focused on identifying SARS-CoV-2-derived svRNAs that evoke IFN-β production.

### SARS-CoV-2 produces excessive amounts of immune-stimulatory small viral RNAs in infected cells

To investigate which svRNA species are involved in IFN-β production during CoV replication, we harvested sRNAs (<200-nt) and lRNAs (>200-nt) from CoV-infected cells. These were then subjected to RNA-sequencing (RNA-seq), as established by others.^27^ The sRNA preparations included a step whereby all 5′ monophosphate host RNAs were first removed by incubation with XRN-1, followed by RppH treatment to enable viral RNAs bearing 5′ PPP to be ligated onto a sequence adapter (Figure 2D). The advantage of the protocol is that it permits the efficient recovery of svRNAs bearing a 5′ PPP from RNA libraries and reduces cellular RNAs bearing other 5′ modifications, such as a 5′ P or a cap, despite also recovering common RNA breakdown products derived from hydrolysis during RNA isolation. In our preliminary analyses, we verified that RppH treatment was essential to read svRNA coding 5′ end viral genomes. Also, XRN-I treatment was not absolutely necessary but acted effectively to enrich read counts of svRNA species without affecting their read profiles (data not shown). We performed two comparative RNAseq analyses, one for human cells (HCT8, MRC5, and Calu-3) and the other for non-human primate cells (Vero).

The sRNA-seq approach provided a high output, similarly for all samples, in the order of 10 million total reads per sample (Table S1). Upon mapping the reads to the human and non-human primate genomes, we found similar read counts for host genes from all the infected cells. We then mapped the reads to the CoV genomes and compared the read counts between them. Total virus reads from SARS-CoV-2 were more abundant than from the HCoVs both in human and Vero cells, up to 27-fold and 4-fold more respectively, despite svRNAs comprising <3% of these libraries (Table S1). The lRNA-seq was also provided in the order of 10 million total reads per sample similarly for all samples (Table S2). However, the relative abundance of virus reads from the lRNA library was less prominent for SARS-CoV-2 than for HCoVs, with up to 9-fold and 2-fold more in human cells and Vero cells, respectively. This implies that SARS-CoV-2 produces svRNAs in greater amounts during RNA synthesis than the HCoVs. Next, we separated the virus reads from the sRNA library by strand specificity and discovered that most reads from all the CoV svRNAs mapped to positive-sense RNA, with >99% occupancy (Figure 2E). These reads were mapped across the entire CoV genome (Figure 2F).

**Figure 3:**
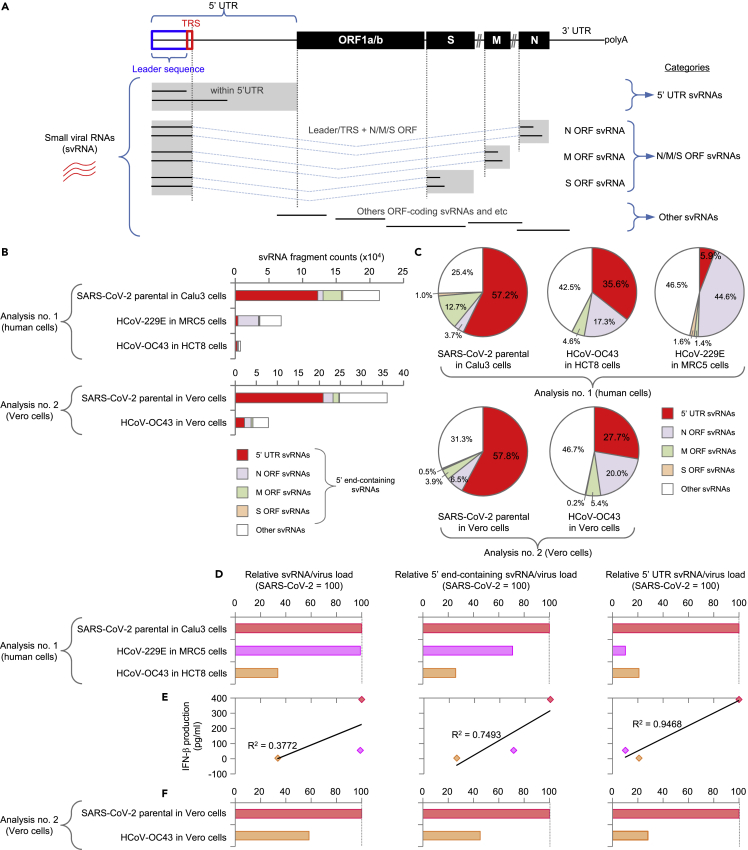
SARS-CoV-2 dominantly produces 5′ UTR svRNAs in infected human and Vero cells (A) Schematic of svRNA fragments produced by CoVs. The svRNAs are divided into three groups: 1) svRNA fragments containing only the 5′ UTRs (termed 5′ UTR svRNAs); 2) svRNA fragments including the leader sequence and TRS jumping to the exact start of major N/M/S ORFs (termed N/M/S ORF coding svRNAs); 3) svRNA fragments harboring leader sequence and TRS fused to other ORFs and all fragments excluding the 5′ UTR (termed other svRNAs). TRS = transcriptional regulatory sequence. (B) Counts of svRNA fragments produced in CoV-infected cells. (C) Fraction of svRNA fragments produced in CoV-infected cells. The numbers (B) and fractions (C) of each svRNA category in the svRNA population of each infection are shown, according to the color legend in the figure. (D and F) Reads of the indicated groups of svRNAs normalized to viral load (lvRNA reads) in human cells (D) and Vero cells (F). (E) Relationship between the normalized svRNA reads (*x* axis) and IFN-β levels (*y* axis) in human cells with linear fitted data.

To characterize the CoV-derived svRNAs, their fragments were categorized into three classes (Figure 3A). First, svRNA fragments present inwards of the 5′ untranslated region (UTR) (here termed “5′ UTR svRNAs”). Second, svRNA fragments containing the leader sequence and transcriptional regulatory sequence (TRS) precisely jumping to the start sites of the major N, M, and S open reading frames (ORFs) (here termed “N/M/S svRNAs”). Third, svRNA fragments harboring the leader sequences and TRS fused to other minor ORFs and all fragments that excluded the 5′ UTR (here termed “other svRNAs”). Notably, counts for the 5′ UTR svRNA fragments were much higher in SARS-CoV-2 than in the HCoVs (Figure 3B), up to 44-fold and 9.6-fold more in human and Vero cells, respectively (Table S3). Patterns of the svRNA classes were dependent on the CoV species but independent of host cell type (Figure 3C). Thus, 5′ UTR svRNAs represented a major fraction of the SARS-CoV-2 svRNAs, but less so in the HCoVs svRNAs, in which N ORF svRNAs were more abundant than for SARS-CoV-2. Nonetheless, a group formed by 5′ UTR svRNAs and N/M/S ORF svRNAs, which constitute most of the 5′ end-containing svRNA species, were always in the majority (about >54% occupancies) in the svRNAs of all these CoVs.

Because the CoVs exhibited distinct virus titers in each human cell, we normalized svRNA reads to the viral load (lvRNA reads) in each cell line for several svRNA groups (e.g. whole svRNA, 5′ end-containing svRNA and 5′ UTR svRNA). The normalized values of 5′ end-containing svRNA and 5′ UTR svRNA from SARS-CoV-2 were higher than those from the HCoVs (Figure 3D), with the most prominent differences observed for 5′ UTR svRNA (4.8-fold and 9.9-fold compared to HCoV-OC43 and -229E, respectively). We then plotted the normalized svRNAs against IFN levels (Figure 3E) and confirmed strong positive correlations for 5′ UTR svRNAs and 5′ end-containing svRNAs (R^2^ = 0.94 and 0.74, respectively), but less so for whole svRNA (R^2^ = 0.37). Similarly, in Vero cells, normalized SARS-CoV-2 svRNA levels were higher than HCoV-OC43, with the greatest difference seen in 5′ UTR svRNA (Figure 3F). These data suggest that greater production of 5′ end-containing svRNAs, in particular 5′ UTR svRNAs, drives the IFN response in SARS-CoV-2-infected cells.

**Figure 4:**
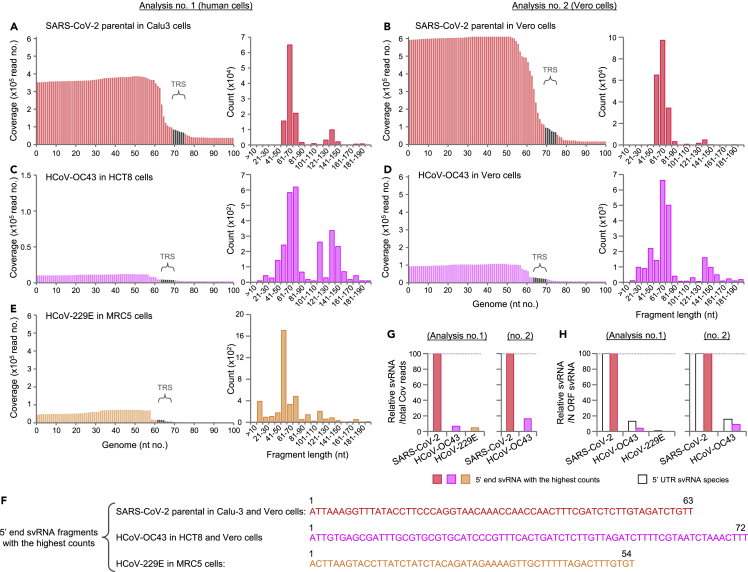
SARS-CoV-2 produces excessive amounts of 5′ UTR svRNAs encoding the precise 5′ end with the representative first 63-nt sequence (A-E, left panels) Read maps of 5′ UTR svRNAs produced by CoVs. Reads were mapped to positive-sense RNA. Read counts were quantified for each nucleotide. (A-E, right panels) Size distribution of 5′ UTR svRNAs. (F) 5′ UTR svRNA fragments with the highest counts in CoV-infected cells. (G) Fragment counts of the most numerous 5′ end svRNAs normalized to virus load (lvRNA reads) in human cells (left) and Vero cells (right). (H) Fragment counts of 5′ UTR svRNA species or the most often 5′ end svRNA normalized to the corresponding N ORF svRNAs as an index of 5′ UTR svRNA production efficiency. The values from the SARS-CoV-2-infected human cells and Vero cells were set to 100%.

The coverage of svRNAs at 5′ end genomes was thus further elucidated. Most strikingly, the high abundance of SARS-CoV-2 svRNAs started from the precise 5′ end (here termed “5′ end svRNAs”) (Figures 4A-4E, left panels). In contrast, substantially less coverage at the 5′ end was seen with the HCoVs, despite the svRNAs mostly harboring exact 5′ termini. The peak sizes of the 5′ UTR svRNA fragments were around 60 to 80-nt (SARS-CoV-2), bimodal at 50 to 80-nt and 140 to 150-nt (HCoV-OC43), and relatively broad at 50 to 80-nt (HCoV-229E) (Figures 4A-4E, right panels). Concordantly, the svRNA fragments with the highest counts had the precise 5′ ends of the CoVs genomes with the first 63-nt for SARS-CoV-2, the first 72-nt for HCoV-OC43, and the first 53-nt for HCoV-229E, with the fragments identical in human and Vero cells (Figure 4F). We normalized fragment counts of the most numerous 5′ end svRNAs to the corresponding viral load and found that the normalized value was >6.1-fold higher for SARS-CoV-2 than for HCoVs (Figure 4G), indicating that the greater 5′ end svRNA production for SARS-CoV-2 was largely due to its higher production rate and not simply due to a much higher viral load in the infected cells. Although the second highest number of reads seemed to be from the latter parts of all CoV genomes which represent N ORF subgenomic (sg) RNAs (Figure 2C), these coverages were mostly due to divided mapping of the latter region of N ORF svRNA fragments grouped to the 5′ end svRNA class. Moreover, fragment counts of 5′ UTR svRNA species and the most often counted 5′ end svRNAs normalized to N ORF svRNAs for SARS-CoV-2 were >6.4-fold and >11.0-fold higher than for the HCoVs, respectively (Figure 4H), implying more marked preferential 5′ end svRNA production rather than leader-primed transcription for SARS-CoV-2, relative to HCoVs. Collectively, these data suggest that svRNA production is specific to the CoV species but not to host cell type, and that svRNAs, particularly 5′ end svRNAs, are produced in extremely large amounts by SARS-CoV-2 relative to HCoVs.

### The first 60-nt sequence of the SARS-CoV-2 5′ end small viral RNAs bearing 5′ PPP and with a duplex structure is responsible for their immune-stimulatory ability

**Figure 5:**
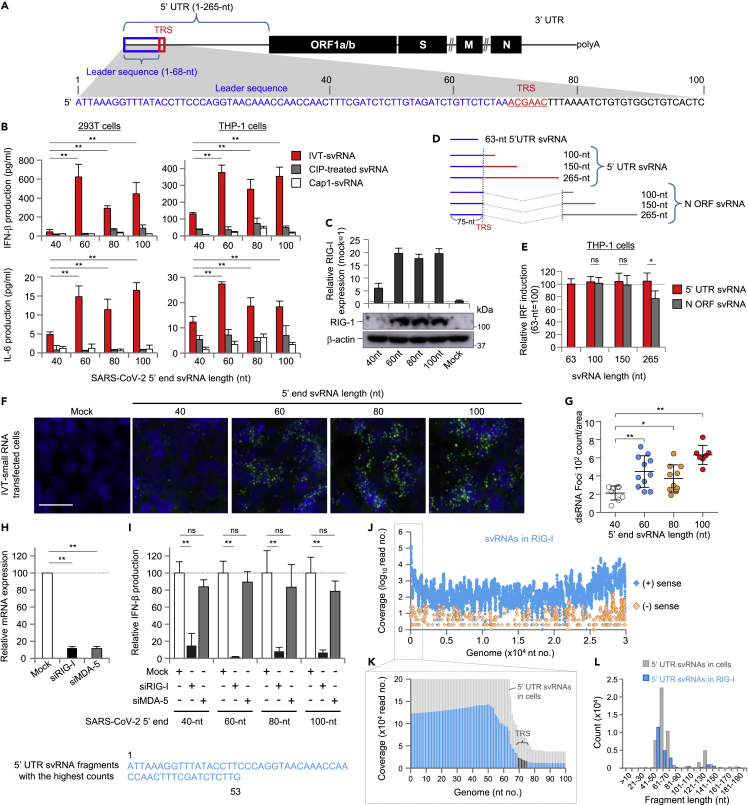
The first 60-nt of 5′ end svRNAs with 5′ PPP and a duplex structure act as RIG-I agonists (A) Sequence of 5′ UTR svRNAs of SARS-CoV-2 used in this study. TRS = transcriptional regulatory sequence. (B) Induction of IFN-β and IL-6 secretion from 293T cells and differentiated THP-1 cells by the transfection of IVT 5′ UTR svRNAs from SARS-CoV-2. Cells cultured in 24-well plates were stimulated by the transfection of 40, 60, 80, and 100-nt IVT 5′ UTR svRNAs without/with CIP treatment or capping (Cap1). At 24 h after stimulation, the supernatants were harvested for ELISA. (C) Immunoblot analysis of RIG-I in lysates from IVT 5′ UTR svRNA-stimulated human cells 24 h post-transfection. Representative images from three independent experiments are shown. β-actin levels are shown as loading controls. (D and E) Stimulatory ability of IRF/type 1 IFN signaling of the 5′ UTR svRNA and N ORF svRNAs with corresponding different lengths. (D) Schematic of IVT 5′ UTR svRNA and N ORF svRNAs with corresponding different lengths used in this study. (E) Induction of IRF/type 1 IFN signaling in THP-1 cells by the transfection of equivalent numbers of copies of the IVT 5′ UTR svRNA and N ORF svRNAs. (F and G) Accumulation of an epitope recognized by the J2 antibody in 293T cells transfected with IVT 5′ UTR svRNA of SARS-CoV-2. At 24 h post-transfection, cells were stained with the J2 anti-dsRNA antibody (green). Nuclei were stained with Hoechst 33,342 (blue). (F) Representative images of dsRNA foci visualized by the J2 antibody. Scale bar = 20 μm. (G) dsRNA foci were quantified using Olympus imaging software from eight randomly selected image fields. (H and I) 293T cells were mock-transfected or transfected with siRNAs against RIG-I (siRIG-I) and MDA-5 (siMDA-5). (H) Levels of RIG-I and MDA-5 mRNA in cells at 24 h post-transfection were measured by real-time RT-qPCR and are expressed relative to mock-transfected cells. (I) Induction of IFN-β secretion from 293T wild-type or RIG-I/MDA-5 knockdown cells by stimulation with IVT 5′ end svRNAs of SARS-CoV-2. At 24 h after mock-transfection or transfection with siRIG-I or siMDA-5, 293T cells were stimulated with IVT 5′ UTR of SARS-CoV-2. The supernatants were harvested for ELISA 24 h after stimulation. IFN-β levels are expressed relative to wild-type cells. Each data point is the mean ± SD from three independent experiments. Statistically significant differences compared to 40-nt IVT svRNA-stimulated cells (B and G), 5′UTR svRNA-stimulated cells (E), or mock-transfected cells (H and I) are determined by ANOVA with Tukey’s multiple comparison test and shown as∗∗p < 0.01 or ∗p < 0.05 (ns = not significant). (J-L) sRNA-seq analysis of sRNAs recognized by RIG-I. Calu-3 cells were infected with SARS-CoV-2 as indicated in the legend to Figure 1. At 72 hpi, sRNA fractions were extracted from total RNAs immunoprecipitated with anti-RIG-I antibody. These immunoprecipitates were evaluated by sRNA-seq. (J) sRNA-seq reads mapped to the SARS-CoV-2 genome. Reads were strand-specifically mapped to the positive-sense (+) RNAs or negative-sense (−) RNAs. Read counts were quantified for each nucleotide of the genome. (K) Read map of 5′ UTR svRNAs from RIG-I. Reads were mapped to the positive-sense RNA. Read counts were quantified for each nucleotide. (L) Size distribution of 5′ UTR svRNAs from RIG-I. A representative svRNA fragment with the highest count is also shown.

To determine the exact sequence of the 5′ end svRNA that is involved in IFN and cytokine production during SARS-CoV-2 replication, *in vitro* transcribed (IVT) RNAs corresponding to the first 40, 60, 80, and 100-nt of the 5′ end of the SARS-CoV-2 genome (Figure 5A) were transfected into 293T cells or into differentiated THP-1 cells, and IFN-β and IL-6 secretion was quantified. IVT RNAs originating from the 60, 80, and 100-nt 5′ ends induced markedly high IFN-β and IL-6 production (Figure 5B), whereas the 40-nt RNA had only a slight effect, correlating with the RIG-I response in cells transfected with IVT RNAs of the corresponding lengths (Figure 5C). Calf intestine alkaline phosphatase (CIP) treatment or capping with a Cap 1 analog resulted in a complete loss of the immune-stimulatory activity, indicating dependence on 5′ PPP for stimulating host antiviral signaling.

We then compared the immune-stimulatory ability of 5′ UTR svRNAs with N ORF svRNAs as another abundant 5′ end-containing svRNA, both of which have the corresponding different lengths but a distinct 3′ region after 75-nt TRS (Figure 5D). Both svRNAs evoked IRF/type 1 IFN signaling at similar levels in THP-1 (IRF reporter) cells (Figure 5E) regardless of 3′ end extension, with the one exception of the N ORF svRNA having somewhat weakened inducing effects at the longest 265-nt. This suggested that 5′ UTR svRNAs have IFN-stimulatory ability equivalent to or even slightly stronger than other 5′ end-containing svRNAs. Thus, these data document that of all these svRNA species, the 5′ end svRNA with a peak length of 63-nt is likely to be the major inducer of the IFN response both qualitatively and quantitatively.

We then hypothesized that SARS-CoV-2 5′ end svRNAs would form high-order RNA structures (Figure S3A), recognized by the J2 antibody that detects duplex RNAs. Indeed, we found that the J2 antibody recognized IVT RNAs of >60-nt in length (Figures 5F and 5G) that formed highly structured epitopes as expected, contributing to the high IFN-β and IL-6 stimulatory activity (Figure 5B). These data show that the induction of IFNs and cytokines by SARS-CoV-2 5′ end svRNAs can be attributed to the first 60-nt sequence that bears 5′ PPP and to the corresponding secondary structure.

We next asked whether exclusively SARS-CoV-2 5′ end sequences had IFN stimulatory ability and not those from other coronaviruses. For this, IVT RNAs corresponding to HCoV-OC43 and HCoV-229E 5′ ends with the same lengths as tested for SARS-CoV-2 were transfected into cells, and IFN-β secretion was quantified. The two HCoV IVT RNAs induced IFN-β production maximally at 60-nt in length, although the peak levels were different among the three CoVs (Figure S3B). These data suggest that the IFN-stimulatory ability of SARS-CoV-2 5′ end svRNAs largely depends on the high quantity of material produced and not on the sequence of the 5′ end region. The 5′ end sequences of CoV species have the highest homology among the genomes^33^ and, indeed, the two HCoV 5′ ends would be expected to form secondary structures similar to the SARS-CoV-2 5′ end (Figure S3C), supporting this notion.

### 5′ end small viral RNAs are recognized by cytosolic retinoic acid-inducible gene-1 but not melanoma differentiation-associated protein 5

SARS-CoV-2 5′ end svRNAs produced in the cytoplasm are thought to be recognized by host RNA sensors. To determine the role of cytosolic RIG-I and MDA-5 in svRNA sensing during SARS-CoV-2 infection, we transfected IVT RNA corresponding to SARS-CoV-2 5′ end svRNAs into 293T RIG-I- or MDA-5-knockdown cells. Silencing was shown to be effective in that specific siRNAs reduced the levels of RIG-1 and MDA-5 mRNAs by 90% compared to mock-transfected cells (Figure 5H). RIG-I silencing reduced IFN-β production to background levels on IVT RNA stimulation, whereas knocking down MDA-5 had little effect (Figure 5I). Furthermore, we sequenced svRNAs co-purified with RIG-I from Calu-3 cells infected with SARS-CoV-2 and found that RIG-I largely recognized the 5′ end svRNAs (Figures 5J-5L). Moreover, this signature was similar to that seen in the cytoplasmic fraction of the infected cells, where the svRNAs mostly start from the exact 5′ end of the genome, on average being 53-nt in length and with positive polarity. These data document that RIG-I is the primary cytosolic sensor of 5′ end svRNAs originating from SARS-CoV-2 and acts cooperatively with MDA-5 mediated by longer viral RNAs to trigger IFN and cytokine responses.

### SARS-CoV-2 5′ end small viral RNAs accumulate in cells at later times after infection *in vitro* and *ex vivo*

**Figure 6:**
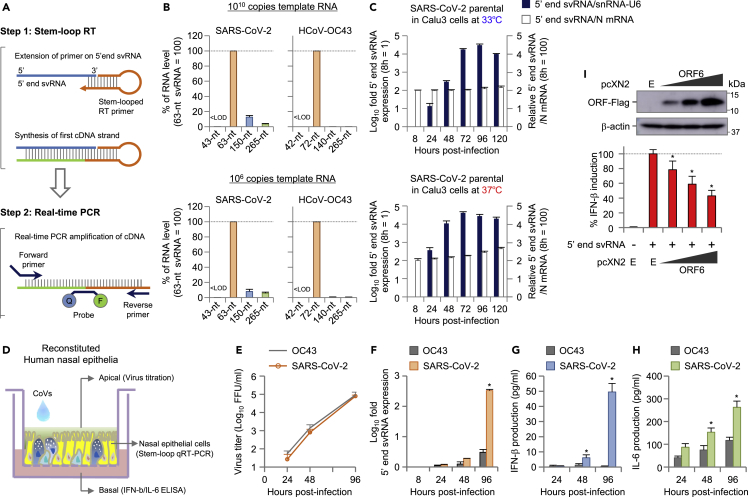
SARS-CoV-2 5′ end svRNAs accumulate in cells at the late stage of infection (A) Schematic of stem-loop RT-qPCR detection of CoV 5′ end svRNAs. cDNA was generated using stem-loop RT primer specific for the representative 5′ end svRNAs with 63-nt for SARS-CoV2 or 72-nt for HCoV-OC43. The qPCR was performed with forward and reverse primers that targeted the 5′ end regions of svRNAs and part of the stem-looped RT primers respectively, as well as the TaqMan MGB probe. (B) Verification of specificity of the stem-loop primers for 5′ end svRNA. The specificity of primer sets specific to the SARS-CoV-2 63-nt end and HCoV-OC43 72-nt end was verified using 5′ end svRNA templates from corresponding CoVs with different 5′ end lengths that were prepared by *in vitro* transcription. Stem-loop qRT-PCR was performed with high dose (10^10^ copies) and middle dose (10^6^ copies) of each template RNA. The specificity of the primer set is shown with respect to the percent of the corresponding RNA templates with 63-nt or 72-nt. LOD, limit of detection. (C) RT-qPCR quantification of 5′ end svRNA levels (left *y* axis) and production efficiency (5′ end svRNA/N mRNA) (right *y* axis) at 8, 24, 48, 72, 96, and 120 hpi in SARS-CoV-2-infected Calu-3 cells at 33°C or 37°C as indicated in the legend to Figure 1. Levels of 5′ end svRNAs were related to those at 8 hpi (set to 1), as calculated by the ΔΔCt method using snRNA-U6 as an endogenous control. Ratios of 5′ end svRNAs to N mRNA were related to those at 8 hpi (set to 100). (D-H) Production of SARS-CoV-2 and HCoV-OC43 5′ end svRNA in *ex vivo* human nasal epithelia reconstituted at the air-liquid interface. (D) Schematic of the experiment. SARS-CoV-2 or HCoV-OC43 were inoculated onto reconstituted human nasal epithelia at an MOI of 0.1, and cultured for 24, 48, and 96 hpi. For collection at the apical side, medium was added for 20 min at 37°C to elute the virus. (E) Virus replication kinetics. Titers of progeny viruses released into apical supernatants were measured by focus-forming assays at the indicated times post-infection. (F) RT-qPCR quantification of intracellular 5′ end svRNA levels at the indicated times post-infection. Stem-loop primers specific to representative SARS-CoV-2 63-nt and HCoV-OC43 72-nt 5′ end svRNAs were used for SARS-CoV-2 and HCoV-OC43, respectively. Levels of 5′ end svRNAs were related to those at 8 hpi as described above. (G and H) IFN-β (G) and IL-6 (H) released from virus-infected cells. At the indicated times post-infection, basal medium was harvested for ELISA. (I) Counteraction between SARS-CoV-2 ORF6 and 5′ end svRNAs. 293T cells were transfected with four amounts of plasmid expressing SARS-CoV-2 ORF6 fused with a FLAG tag. At 24h post-transfection, the cells were stimulated with 63-nt IVT 5′ end svRNAs from SARS-CoV-2. At 48 h post-transfection, cells and supernatants were harvested for Western blotting and ELISA. For Western blotting one representative result of three independent experiments is shown. For ELISA, the value of the IVT-RNA-stimulated and empty-plasmid-transfected cells was set to 100%. Statistically significant differences compared to values at 8 hpi (F), 24 hpi (G and H) and empty plasmid-transfected cells (I) are determined by ANOVA with Tukey’s multiple comparison test and shown as ∗p < 0.01.

To determine 5′ end svRNA biogenesis in infected cells, the kinetics of SARS-CoV-2 5′ end svRNA production were studied in Calu-3 cells under the same conditions as in Figure 1. Quantitative reverse transcription-polymerase chain reactions (qRT-PCRs) were performed using stem-loop RT primers specific for the most common svRNAs having 63-nt for SARS-CoV-2 or 72-nt for HCoV-OC43 (Figure 6A). The specificity of the primer sets was confirmed using IVT RNA templates with different 5′ end lengths (Figure 6B). This showed that the SAR-CoV-2 63-nt-specific primer set could detect <3% of 43-nt and 265-nt 5′ end svRNAs, with minimal detection at <10% of the 150-nt counterpart. The HCoV-OC43 72-nt-specific primer set could also detect <2% of 5′ end svRNAs having other lengths. These data indicated that these stem-loop qRT-PCRs were able to distinguish different lengths of 5′ end-containing RNAs from the two CoVs (i.e. 5′ end svRNA vs. genomic/subgenomic RNAs) at a similar level. The levels of svRNAs were normalized to levels of snRNA-U6, and values were expressed relative to the earliest sampling time (8 hpi). As expected, only minimal amounts of the 5′ end svRNAs were detected at 8 hpi (Figure 6C, left *y* axis), in agreement with the lack of significant IFN-β secretion at this time. The svRNAs progressively accumulated up to 96 hpi at 33°C and 72 hpi at 37°C, with a >5 log increase compared to levels at 8 hpi. We also calculated the ratio of 5′ end svRNA to N mRNA as a representative viral mRNA over the time course of SARS-CoV-2 infection. We expected this ratio would be increased at later stages of infection, if 5′ end svRNA production results from RNA degradation. However, the ratios were constant throughout the infection period (Figure 6C, right *y* axis), showing that 5′ end svRNA level was proportional to regularly transcribed viral mRNA levels. Thus, it is justified to propose that 5′ end svRNAs are synthesized by aberrant transcription rather than RNA degradation. These data suggest that 5′ end svRNAs arise at low levels early in the viral life cycle and accumulate in infected cells over time, reaching high levels at later times after infection.

To further define the profile of SARS-COV-2 5′ end svRNA production *ex vivo*, reconstituted human nasal epithelia were infected with SARS-CoV-2 or HCoV-OC43 at an MOI of 0.1 (Figure 6D). The kinetics of production of progeny viruses and 5′ end svRNAs and expression profiles of IFN-β and IL-6 were compared between the two viruses. SARS-CoV-2 and HCoV-2 showed similar replication kinetics in the reconstituted human epithelia, yielding >4 log FFU/mL at 96 hpi (Figure 6E). In contrast, there was a clear difference between them in terms of 5′ end svRNA production; thus, SARS-CoV-2 accumulated to a high degree at 96 hpi, whereas HCoV-OC43 produced lesser amounts throughout the culture period (Figure 6F). These findings are in accord with the marked elevation of the IFN-β level at 96 hpi (Figure 6G) and to some extent with the gradual increase in the IL-6 level at 48 and 96 hpi (Figure 6H). These *ex vivo* data further support the interpretation that SARS-CoV-2 svRNAs accumulate excessively during replication in human cells, compared with endemic HCoVs.

### Stoichiometric balance of SARS-CoV-2 interferon antagonists and 5′ end small viral RNAs determines the host interferon response

SARS-CoV-2 encodes numerous accessory proteins (e.g. ORF6) that act as IFN antagonists.^34^^,^^35^ These are initially translated from the viral genomic RNA after virus entry to swiftly inhibit type 1 IFN production. In contrast, as shown in Figure 6C, svRNAs accumulate in infected cells at later times after infection. We thus hypothesized that IFN activation mediated by accumulated 5′ end svRNAs overcomes the antagonistic ability of the accessory proteins and drives later IFN production. To elucidate counteractions between the IFN antagonists and 5′ end svRNA agonists in host cells, we stimulated 293T cells ectopically expressing SARS-CoV-2 ORF6 with IVT 5′ end svRNAs from SARS-CoV-2. ORF6 dose-dependently suppressed IFN induction mediated by IVT 5′ end svRNAs (Figure 6I). Thus, these data demonstrate that the stoichiometric balance between IFN antagonists and IFN-stimulatory 5′ end svRNAs determines the IFN response in host cells, consistent with our hypothesis.

### The SARS-CoV-2 Delta variant produces 5′ end small viral RNAs in infected cells at levels similar to the parental virus

Delta variants reportedly have a higher replication efficiency in human airway epithelia,^36^ indicating a better fitness in human cells. Thus, we investigated whether their fitness in humans had resulted in a reduced production of erroneous svRNAs in infected human cells. The Delta strain (termed Delta) efficiently infected Calu-3 cells and produced moderately larger amounts of viral progeny with slightly more rapid replication kinetics than the parental strain (Figures S4A and S4B). This was accompanied by induction both of IFN-β and RIG-I at either temperature (Figures S4C-S4E) as seen in the parental strain.

sRNA-seq generated a high output of reads per sample (Table S4) and showed that svRNAs from Delta were more abundant than from the HCoVs, despite the svRNAs being represented to only a minor degree in the library (<0.63%). Again, a large majority of the Delta reads mapped to positive-sense RNA with the 5′ end having the highest coverage (Figures S4F and S4G). The Delta 5′ end svRNAs were the major svRNA categories mostly having the exact 5′ end (Figure S4H) and produced at levels similar to the parental strain (Table S5). svRNA fragment patterns were identical to the parental strain with a peak size of 63-nt (Figure S4I). qRT-PCR using the above-mentioned looped RT primers revealed that 5′ end svRNAs accumulated in infected human cells with similar kinetics to the parental strain (Figure S4J). Delta also exhibited 5′ end svRNA production comparable to the parental strain in Vero cells despite its moderately slower replication kinetics (Figures S4K and S4L). These data show that Delta has evolved in humans without correcting the svRNA production in human cells.

### The SARS-CoV-2 Omicron variants BA.1 and BA.2 produce less 5′ end small viral RNAs in infected cells than the parental virus

**Figure 7:**
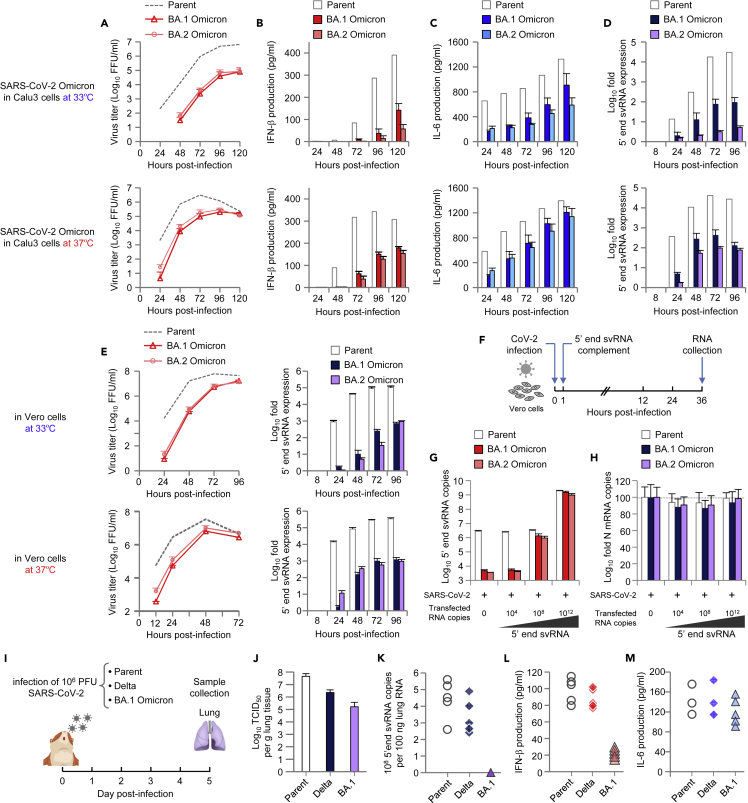
SARS-CoV-2 Omicron BA.1 and BA.2 produce less 5′ end svRNAs and are less immunostimulatory *in vitro* and *in vivo* (A) Replication kinetics of the SARS-CoV-2 Omicron (BA.1 and BA.2) variant in human Calu-3 cells. Cells were infected at an MOI of 0.001 and incubated either at 33°C or 37°C. Titers of viral progeny were measured by FFU assays at the indicated times post-infection. (B and C) Induction of IFN-β (B) and IL-6 (C) secretion by Omicron-infected Calu-3 cells. At the indicated times post-infection, the supernatants were harvested for ELISA. (D) RT-qPCR quantification of 5′ end svRNA levels at 8, 24, 48, 72, 96, and 120 hpi in Omicron-infected Calu-3 cells at 33°C or 37°C as indicated in the legend to Figure 1. Levels of 5′ end svRNAs were related to those at 8 hpi, as calculated by the ΔΔCt method using snRNA-U6 as an endogenous control. (E) Vero cells were infected with the SARS-CoV-2 Omicron (BA.1 and BA.2) at 33°C or 37°C as indicated in the legend to Figure 1. (left panel) Replication kinetics of Omicron. (right panel) RT-qPCR quantification of 5′ end svRNA levels at 8, 24, 48, 72 and 96 hpi in Vero cells infected with Omicron. Levels of 5′ end svRNAs were related to those at 8 hpi, as calculated by the ΔΔCt method using snRNA-U6 as an endogenous control. Each data point is the mean ± SD from three independent experiments. (F-H) 5′ end svRNA complementation assay. (F) Illustration of the 5′ end svRNA complementation assay schedule. Vero cells were infected with SARS-CoV-2 parental virus and Omicron variants (BA.1 and BA.2) at an MOI of 0.001. After 1 h adsorption and washing, cells were transfected with different amounts of representative 5′ end svRNA having 63-nt. Intracellular RNAs were collected after an additional incubation for 36 h at 37°C. (G) RT-qPCR quantification of 5′ end svRNA levels using the same stem-loop RT primer for the 63-nt 5′ end svRNA. (H) RT-qPCR quantification of N mRNA levels. Levels of N mRNAs were related to those of mock transfection. Each data point is the mean ± SD from three independent experiments. (I-M) Infection of hamsters with SARS-CoV-2 parental virus, Delta or BA.1 Omicron variants. (I) Illustration of the SARS-CoV-2 infection schedule in the Syrian hamster model with 1×10^6^ plaque-forming units (PFU) virus inoculum. (J) Viral titers in the lungs of infected hamsters (5 animals per group) at 5 dpi. (K) RT-qPCR quantification of 5′ end svRNA levels in lungs of hamsters at 5 dpi using the same stem-loop RT primer for the 63-nt 5′ end svRNA. (L and M) Responses of IFN-β (L) and IL-6 (M) in the lungs of hamsters infected with parental virus, Delta, and Omicron variants at 5dpi.

Omicron variants have emerged with multiple mutations and reportedly have greater transmissibility at the same time as reduced pathogenicity.^2^ While the original Omicron lineage BA.1 had become dominant in many countries, the latest Omicron lineage BA.2 has outcompeted previous variant lineages (including Delta and BA.1), becoming dominant worldwide, as of May 2022. We investigated whether these variants produce less aberrant 5′ end svRNAs in human cells. Compared with the parental strain, the Omicron variants BA.1 and BA.2 (termed BA.1 and BA.2) replicated less well in Calu-3 cells as reported previously^37^ (Figure 7A). IFN-β expression was markedly attenuated, although finally achieving a substantial level at 120 hpi at 37°C (Figure 7B), whereas IL-6 expression was less reduced in Omicron-infected cells (Figure 7C). These correlated with only a modest accumulation of 5′ end svRNAs from BA.1 and BA.2 (Figure 7D). In Vero cells, Omicron (BA.1 and BA.2) again showed slower replication kinetics and markedly less accumulation of 5′ end svRNAs (Figure 7E). Thus, less 5′ end svRNA during Omicron infection was associated with decreased virus production.

Because Omicron (BA.1 and BA.2) produced markedly lower amounts of 5′ end svRNAs during replication than the parental virus, we performed a 5′ end svRNA complementation assay and titrated different amounts of the representative 5′ end svRNA having 63-nt during Omicron (BA.1 and BA.2) replications in Vero cells (Figure 7F). Transfection with 10^8^ copies of 5′ end svRNA restored intracellular 5′ end svRNA levels similar to the parental virus, whereas 10^12^ copies exceeded inherent 5′ end svRNA levels resulting from infection with the parental virus (Figure 7G). Nonetheless, these supra-adequate and excessive amounts had little impact on the viral load (N mRNA copies) of Omicron (BA.1 and BA.2) or even of the parental virus (Figure 7H). These data indicate that 5′ end svRNAs were not in-demand materials for SARS-CoV-2 replication *per se*, but seemed to exclusively stimulate an excessive immune response to virus infections.

### SARS-CoV-2 produces 5′ end small viral RNAs during virus replication in hamsters

To address whether 5′ end svRNAs are produced on SARS-CoV-2 infection *in vivo*, an established hamster infection model was used (Figure 7I). Infection in this species does not result in severe symptoms or ARDS, but represents a reproducible COVID-19 model with moderate pneumonia and inflammatory infiltrates in the lung.^38^^,^^39^ We infected hamsters with SARS-CoV-2 parental, Delta, and BA.1 Omicron strains at the same inoculation titers and sampled lungs and serum at 5 dpi. Viral titers were measured in the lungs and 5′ end svRNA levels in the lungs and sera by stem-loop RT-PCR for the 63-nt 5′ end svRNAs described above. All infected hamsters exhibited sufficiently high virus titers in the lungs with >5 log TCID50/g tissue (Figure 7J), although Delta had a slightly lower titer and Omicron had a moderately lower titer than the parental strain, consistent with prior reports for Omicron BA. 1.^40^ The 5′ end svRNAs were present in all infected lung at levels of >8 log copies/100 ng RNA in all hamsters infected with parental and Delta strains (Figure 7K). In marked contrast, these were below the level of detection in lungs infected with Omicron, which is in accord with the marked elevation of the IFN-β level in hamsters infected with parental and Delta (Figure 7L) and less with the increase in the IL-6 level in those infected with all the three virus strains (Figure 7M). These data are consistent with the results from cultured human and Vero cells.

We analyzed the genetic conservation of several replication-associated viral proteins among the SARS-CoV-2 parental virus, Delta, and Omicron (BA.1 and BA.2) variants used in this study. Aligning amino acid sequences of these viral proteins showed that NSP14-I42V is the only mutation shared by Omicron BA.1 and BA2 compared to the parental virus and Delta (Table S6). The almost identical amino acid sequences among the SARS-CoV-2 strains with only a single mutation (I-to-V substitution) suggest that lower production of 5′ end svRNAs by Omicron (BA.1 and BA.2) was not due to their replication-associated viral proteins, but probably due to Spike mutations conferring their characteristic *in vitro* and *in vivo* replication properties. Indeed, the replication kinetics of Delta and Omicron (BA.1 and BA.2) observed in the present study correspond to those of pseudoviruses carrying the corresponding SARS-CoV-2 Spikes quite well.^37^ These findings suggest that SARS-CoV-2 has not yet matched replication-associated genes in human cells, with its inherent svRNA productivity being unchanged. Taken together, these data indicate that immune-stimulatory 5′ end svRNA levels are variable across the SARS-CoV-2 variant lineages and are associated primarily with viral replication properties, which are largely attributed to Spike characteristics in infected cells *in vitro* and *in vivo*, at least regarding SARS-CoV-2 variant evolution to date.

## Discussion

Here, we found that SARS-CoV-2 generates significantly higher levels of svRNAs that originate from the exact 5′ ends in infected human and Vero cells than do HCoV-OC43 and HCoV-229E. The 5′ end svRNAs activated RIG-I and led to high expression of IFN-β and IL-6, both of which can contribute to the hyperinflammatory responses observed in severe COVID-19.^3^^,^^41^ Most studies investigating this issue have used total RNA libraries for RNA-seq to map viral RNA transcripts, where sRNAs species have rarely been captured. In contrast, RNA preparation technique we opted for here, which was previously established by others,^27^ included enzyme treatments whereby only sRNAs (<200-nt) bearing 5′ PPP were enriched, which enabled us to capture unexpectedly large amounts of svRNAs bearing 5′ PPP by RNA-seq. Although the enzyme treatments might potentially affect the integrity of svRNA species (e.g. by RNA degradation), these treatments were applied uniformly to all the RNA samples tested here, which should not bias the difference in svRNA productivity among CoVs in this study. Therefore, our study showed substantial utility of this method to faithfully detect 5′ termini of viral RNA genomes.

Our analyses resulted in the discovery of svRNAs derived from SARS-CoV-2. There is growing evidence that support the generation of svRNAs originating from RNA viruses including influenza virus,^23^ HCV, Dengue virus, West Nile virus,^42^ and SARS-CoV-1.^43^ Most svRNAs, recovered from SARS-CoV-2-infected cells, were the precise 5′ end of the positive-sense genome under uncapped conditions. The strand specificity indicated that they were synthesized on an antigenomic intermediate template during replication. One could envisage that the svRNAs were RNA degradation products. However, the 5′ end svRNA/viral mRNA profile was constant during SARS-CoV-2 infection, implying production by aberrant replication but not RNA degradation. Instead, we found that small numbers of svRNAs with random sizes mapped equally across the entire genome of the CoVs investigated here, implying that these random svRNAs are viral RNA breakdown products and support the notion that the 5′ end svRNAs were produced during viral replication. The production of similar 5′ terminal viral RNAs was first described during influenza virus replication^23^^,^^44^ despite their shorter length (22-27-nt). The influenza svRNAs correspond to the exact 5′ end of the RNA genome and are supposedly involved in transcription-to-replication switching, but not IFN induction. In contrast, our results suggest that SARS-CoV-2 5′ end svRNAs can drive robust immune responses. This may be explained by the distinct 5′ end svRNAs spectrum of length (average 25-nt in influenza viruses vs. 65-nt in SARS-CoV-2), enabling SARS-CoV-2 5′ end svRNA binding to RIG-I. Indeed, whereas 5′ end svRNAs of SARS-CoV-2 ≥60-nt in length effectively activated the IFN response, shorter 40-nt 5′ end svRNAs had a lesser ability to do so despite the presence of the SL1 structure (Figure 5B). Another difference is that CoVs replicate in the cytoplasm, whereas influenza viruses replicate in the nucleus, so cytosolic RNA sensors barely have access to intracellular influenza svRNAs.

5′ end svRNAs from the CoVs detected in this study mostly corresponded to the leader sequence and ended around the TRS that drives discontinuous transcription. The leader sequence is located at the 5′ end of all viral RNAs, not only in the viral genome, but also in the subgenomic RNAs, which make it the most abundant viral RNA sequence present during infection. However, interestingly, these sequences were found as small RNA species, instead of being fused to longer RNAs (genomic and subgenomic RNAs) and they accumulated in infected cells under uncapped conditions. Most probably, the 5′ end svRNAs result either from premature termination caused by non-processive transcription of the 3′ end of the intermediate, or mature termination followed by dysregulated stacking before utilizing discontinuous transcription. Many details regarding the biogenesis and function of the separated CoV 5′ end svRNAs in the virus life cycle remain to be determined. Unfortunately, our current data can not completely exclude the possibility that CoV 5′ end svRNAs are degraded by-products of replication. Nonetheless, regardless of how they are generated, the presence of abundant cytoplasmic SARS-CoV 5′ end svRNAs is, at least partially, associated with host immune activation in the late stage of the infection.

In replicating influenza A virus, small aberrant viral RNAs containing both the 5 and -3′ ends of viral RNAs were shown to activate RIG-I^27^; erroneous or dysregulated replication by avian influenza virus causes high production of these aberrant RNAs, underlying the high IFN and cytokine inductions in mammals. Similarly, SARS-CoV-2 probably has not completed human adaptation, whereas endemic HCoVs are fully human-adapted viruses. Consistent with its poorer human adaptation, highly abundant svRNAs were present in human/Vero cells or reconstituted human airway epithelia infected with SARS-CoV-2 compared to those infected with HCoV-OC43 and/or HCoV-229E. The production of 5′ end svRNA appeared to correlate with virus replication. In many cases, greater virus replication was accompanied by higher 5′ end svRNA production, as was seen with the SARS-CoV-2 parental virus and Delta. As an exception, in human airway epithelia *ex vivo*, SARS-CoV-2 and HCoV-OC43 exhibited comparable virus production, but 5′ and svRNA production was significantly great for SARS-CoV2. Importantly, each CoV eventually spread the infection to almost 100% of the cell population and reached a plateau. This implies that SARS-CoV-2 may require more virus particles to propagate, whereas the HCoVs disseminate more efficiently with fewer virus particles. This may explain why SARS-CoV-2 robustly produces 5′ end svRNA during replication, implying a possible association with host adaptability. However, CoV evolutionary modification of 5′ end svRNA production requires further in-depth monitoring to confirm an association of this property of SARS-CoV-2 with changes in disease course.

CoVs replicate in the cytoplasm of host cells, wherein cytosolic RNA sensors, including RIG-I and MDA-5, are mostly responsible for monitoring viral RNAs. RIG-I preferentially recognizes short-structured RNAs with 5′ PPP ends, whereas MDA-5 ligands are much less well-characterized and are presumed to be long-structured RNAs, with no requirement for 5′ PPP.^45^ Thus, SARS-CoV-2 5′ end svRNAs that possess 5′ PPP and duplex structures are ideal RIG-I ligands and, consistent with this, they activate RIG-I-dependent IRF/type 1 IFN signaling pathways. In addition to the avian influenza svRNAs,^27^ RIG-I was also shown to sense leader-containing short RNAs of other positive-sense RNA viruses such as flaviviruses,^45^ where RIG-I recognized the 5′ PPP ends of nascent transcripts before capping, inducing IFN secretion. Both RIG-I and MDA5 mediate innate immune responses during SARS-CoV-2 infection,^11^^,^^12^ although MDA5 is regarded as a central mediator of IFN production.^13^^,^^14^ These results support the notion that SARS-CoV-2 5′ end svRNAs are specific or at least preferred RIG-I ligands, and likely contribute to excessive immune responses, along with longer viral RNAs that activate MDA-5.

CoVs have developed diverse strategies to counteract IFN responses, in particular the type 1 IFN pathway.^10^^,^^46^^,^^47^ Numerous nonstructural proteins and accessory ORF proteins from various CoVs were shown to prevent type 1 IFN induction and its downstream STAT1 signaling pathway in human cells.^48^^,^^49^^,^^50^ After infection by SARS-CoV-2, viral positive-sense genomic RNA facilitates rapid translation of these proteins, which in turn antagonizes viral RNA-induced RIG-I-IFN signaling. The antagonistic capability of CoV proteins was shown to be more selective for type 1 IFN signaling than NF-κΒ signaling during CoV infections.^10^^,^^35^^,^^46^ This may promote the activation of RIG-I-NF-κΒ signaling, leading to the release of other cytokines in the absence of IFN responses at early infection stages, as seen with HCoV-229E infection in the present study. Compared to the endemic HCoVs, SARS-CoV-2 infection results in the marked accumulation of 5′ end svRNAs and reaches higher levels at later stages of infection; the threshold for RIG-I activation can then be achieved by overcoming the antagonistic ability of viral defense proteins, which in turn drives exuberant IFN production and multiple ISGs. Therefore, the stoichiometric balance between antagonistic viral proteins and agonistic 5′ end svRNAs may explain, at least to some extent, the immune-pathology observed in patients with severe COVID-19.^16^^,^^51^ Also, our data that the production of svRNAs and the corresponding IFN response were variable among CoV strains suggest that lower svRNA production during replication may correlate with the weaker immune response seen in CoVs with lower pathogenicity, However, it should be noted that IFN responses involve multiple mechanisms and result not only from svRNA production but also from replication rate and the success of viral strategies to attenuate the immune response.

The 5′ end sequences of CoVs have the highest similarity among any part of their genomes.^33^ RNA-based antisense therapy is now widely used. Our studies thus extend the understanding of SARS-CoV-2 immunopathology and shed light on the design of drug targets against COVID-19 and future emerging CoV variants of concern.

### Limitations of the study

Our study has several limitations. First, although we found that SARS-CoV-2-derived svRNAs stimulate RIG-I whereas SARS-CoV-2 infection *per se* activates both RIG-I and MDA5 using several human cell lines, studies by others have shown that whether SARS-CoV-2 infection primarily or specifically elicits either RIG-I or MDA5 may depend on the cell type used in the experiments.^11^^,^^12^^,^^13^^,^^14^ Therefore, rigorous follow-up studies are needed to validate svRNA specificity for RIG-I activation using more different cell types and to provide conclusive evidence that svRNAs directly bind to RIG-I, even though our RIP assay indicated their direct or indirect association in SARS-CoV-2 infected cells. Second, our study examines only hamster infection, which is a model of SARS-CoV-2 infection with only mild-to-moderate symptoms. Thus, further studies using clinical samples from patients with severe COVID-19 are needed to validate the association of the 5′ end svRNA production *in vivo* with disease aggravation.

## STAR★Methods

### Key resources table

**Table:** 

REAGENT or RESOURCE	SOURCE	IDENTIFIER
**Antibodies**		

SARS-CoV-2 (COVID-19) nucleocapsid antibody	GeneTex	Cat# GTX135357; RRID: AB_2868464
Human coronavirus (HCoV-229E) Nucleocapsid Antibody, Rabbit PAb	SinoBiological	Cat# 40640-T62
Human coronavirus (HCoV-OC43) Nucleocapsid Antibody, Rabbit PAb	SinoBiological	Cat# 40643-T62
Rig-I (D14G6) Rabbit mAb	Cell Signaling Technology	Cat# 3743; RRID: AB_2269233
MDA-5 (D74E4) Rabbit mAb	Cell Signaling Technology	Cat#5321; RRID: AB_10694490
Anti b-actin, Monoclonal Antibody	FUJIFILM WAKO	Cat#010-27841; RRID: AB_2858279
SARS-CoV-2 (COVID-19) Spike antibody	GeneTex	Cat# GTX135356; RRID: AB_2887482
Mouse anti double-stranded RNA (J2)	Jena Bioscience	Cat# 10010500; RRID: AB_2922431
Monoclonal ANTI-FLAG® M2 antibody produced in mouse	Sigma-Aldrich	Cat# F3165; RRID: AB_259529
Anti-CD63 (LAMP-3) mAb	MBL	Cat# MEX002-3
Goat anti-Rabbit IgG (H+L) Highly Cross-Adsorbed Secondary Antibody, Alexa Fluor 488	Thermo Fisher Scientific	Cat# A-11034; RRID: AB_2576217
Goat anti-Mouse IgG (H+L) Highly Cross-Adsorbed Secondary Antibody, Alexa Fluor 488	Thermo Fisher Scientific	Cat# A-11029; RRID: AB_2534088
Peroxidase Donkey Anti-Rabbit IgG (H+L)	Jackson Immuno Research	Cat# 711-035-152; RRID: AB_10015282
Peroxidase Donkey Anti-Mouse IgG (H+L)	Jackson Immuno Research	Cat# 715-035-150; RRID: AB_2340770

**Bacterial and virus strains**		

SARS-Cov-2 parental strain	NIID	LC522975
SARS-Cov-2 delta strain	NIID	EPI_ISL_2158617
SARS-CoV-2 BA.1 Omicron strain	NIID	EPI_ISL_7418017
SARS-CoV-2 BA.2 Omicron strain	NIID	EPI_ISL_9595859
HCov-OC43	ATCC	VR-1558
HCov-229E	ATCC	VR-740

**Chemicals, peptides, and recombinant proteins**		

TransIT®-LT1 Reagent	Mirus	Cat# MIR 2304
TransIT-TKO® Transfection Reagent	Mirus	Cat# MIR 2154
TransIT®-mRNA Transfection Kit	Mirus	Cat# MIR 2225
XRN-I	New England Biolabs	Cat# M0338S
RNA 5′ Pyrophosphohydrolase (RppH)	New England Biolabs	Cat# M0356
Xba I	TAKARA Bio	Cat# 1093A
Hind III	TAKARA Bio	Cat# 1060A
Ribo m7G Cap Analog	PROMEGA	Cat# P171A
mRNA Cap 2′-O-Methyltransferase	New England Biolabs	Cat# M0366S
Dynabeads™ Protein G for Immunoprecipitation	Thermo Fisher Scientific	Cat# 10003D
ExoQuick-TC	System Bioscience	Cat# EXOTC10A
Phorbol 12-myristate 13-acetate	Sigma-Aldrich	Cat# P8139
Geneticin™ Selective Antibiotic (G418 Sulfate)	Thermo Fisher Scientific	Cat# 10131035
Quick CIP	New England Biolabs	Cat# M0525

**Critical commercial assays**		

Human IFN-beta Quantikine ELISA Kit	R&D Systems	Cat# DIFNB0
Human IL-6 Quantikine ELISA Kit	R&D Systems	Cat# D6050
miRNeasy Mini Kit	QIAGEN	Cat# 217004
QUANTI-Luc	InvivoGen	Cat# rep-qlc1
QIAquick PCR Purification Kit	QIAGEN	Cat# 28106
T7 RiboMAX™ Express Large Scale RNA Production System	PROMEGA	Cat# P1320
RNeasy Mini Kit	QIAGEN	Cat# 74104
TaqMan™ MicroRNA Reverse Transcription Kit	Thermo Fisher Scientific	Cat# 4366596
TaqMan™ Fast Advanced Master Mix	Thermo Fisher Scientific	Cat# 444557
NEBNext Multiplex Small RNA Library Prep Set for Illumina	New England Biolabs	Cat# E7300
QIAquick Gel Extraction Kit	QIAGEN	Cat# 28704
SMART-Seq® HT Kit	Takara Bio	Cat# 634455
Nextera XT DNA Library Prep Kit (96 Samples)	Illumina	Cat# FC-131-1096
TaqMan Gene Expression Assay for RIG-I	Thermo Fisher Scientific	Hs01061436_m1
TaqMan Gene Expression Assay for MDA5	Thermo Fisher Scientific	Hs00223420_m1
TaqMan Gene Expression Assay for 18s ribosomal RNA	Thermo Fisher Scientific	Hs99999901_s1
Custom Taqman Small RNA Assays for SARS-CoV-2 5′ end svRNA	Thermo Fisher Scientific	CTEPTH7
Custom Taqman Small RNA Assays for Hcov-OC43 5′ end svRNA	Thermo Fisher Scientific	CTFVK34
QuantiTect Probe RT-PCR Kits	QIAGEN	Cat# 204345
Taqman MicroRNA Assay for snRNA-U6	Thermo Fisher Scientific	Assay ID 001973
Silencer Select Pre Designed siRNA for MDA5	Thermo Fisher Scientific	s34499
Nori Hamster IL-6 ELISA Kit	Genorise	GR172141
Nori Hamster Interferon beta ELISA Kit	Genorise	GR172105

**Experimental models: Cell lines**		

Human 293T cells	RIKEN BRC	RCB2202
Monkey Vero cells	RIKEN BRC	RCB0001
Human Calu-3 cells	ATCC	HTB-55
Human MRC5 cells	ATCC	CCL-171
Human HCT8 cells	ATCC	CCL-244
Human THP-1 cells	JCRB Cell Bank	JCRB0112.1
Monkey Vero/TMPRSS2 cells	JCRB Cell Bank	JCRB1819
MucilAir nasal epithelia	Epithelix	EP01MD

**Experimental models: Organisms/strains**		

Syrian Hamster (four-week-old female)	SLC Japan	N/A

**Oligonucleotides**		

siRNA targeting sequence: RIG-I GACUAGUAAUGCUGGUGUAUU	FASMAC	N/A

**Recombinant DNA**		

pcXN2 plasmid	Niwa et al., 1991 PMID: 1660837	N/A
pUC18 plasmid	Takara Bio	Cat# 3218
SARS-CoV-2 ORF6	Eeurofins	N/A

**Software and algorithms**		

GraphPad Prism 6	GraphPad	https://www.graphpad.com
Cutadapt version 3.4	Marcel et al.^52^	https://cutadapt.readthedocs.io/en/v3.4/index.html
HISAT2 version 2.1.0	Kim et al., 2019 PMID: 31375807	http://daehwankimlab.github.io/hisat2/download/
Illumina RTA3 v3.4.4	Illumina, Inc.	https://jp.support.illumina.com/npi/novaseq-6000/downloads.html

### Resource availability

#### Lead contact

Further information and requests for resource and regent should be directed to and will be fulfilled by the lead contact, Yohei Watanabe (nabe@koto.kpu-m.ac.jp).

#### Materials availability

All requests regenerated in this study are listed in the key resources table and are available from the lead contact with a completed Materials and Transfer Agreement.

### Experiment model and subject details

#### Hamster

The hamster experiments were approved by the Institutional Committee of Laboratory Animal Experimentation of Research Institute for Microbial Diseases, Osaka University (R02-08-0). All efforts were made during the study to minimize animal suffering and to reduce the number of animals used in the experiments.

#### Cell lines

293T cells (human embryonic kidney cell line) and Vero cells (African green monkey kidney cell line) were obtained from the RIKEN BioResource Center Cell Bank. Calu-3 cells (human bronchial epithelial cell line), MRC5 cells (human fetal lung fibroblast cell line) and HCT8 cells (human rectal adenocarcinoma cell line) were obtained from the American Type Culture Collection (ATCC). THP-1 cells and Vero/TMPRSS2 cells^53^ were obtained from the Japanese Collection of Research Bioresources Cell Bank. 293T, Calu-3 and MRC5 cells were maintained in Dulbecco’s modified Eagle’s Medium (DMEM) containing 10% fetal calf serum (FCS). HCT8 and THP-1 cells were maintained in RPMI-1640 medium with 10% FCS. Vero cells and Vero/TMPRSS2 cells were maintained in DMEM containing 10% FCS. 1 mg/mL G418 (Invivogen) was added to the growth medium for Vero/TMPRSS2 cells. THP-1-dual reporter cells, THP-1-dual KO-RIG-I cells and THP-1-dual KO-MDA5 cells were purchased from InvivoGen and maintained according to the manufacturer’s instructions.

#### Primary cell culture

Human *ex vivo* reconstituted nasal epithelia (from a 43-year-old female Caucasian) was purchased from Epithelix and maintained at an air-liquid interface with provided culture medium, according to the manufacture’s instructions. All samples purchased from Epithelix have been obtained with informed consent.

### Method details

#### Viruses

The parental strain JPN/TY/WK-521/2020 (GenBank: LC522975), the Delta (B.1.617.2 lineage) strain Japan/TY11-927/2021 (GISAID: EPI_ISL_2158617), the Omicron (B.1.1.529.1 lineage/BA.1) strain Japan/TY38-873/2021 (GISAID: EPI_ISL_7418017) and the Omicron (B.1.1.529.2/BA.2) strain Japan/TY40-385/2022 (GISAID: EPI_ISL_9595859) of SARS-CoV-2 were kindly provided by the National Institute of Infectious Diseases, Tokyo Japan. HCov-OC43 (ATCC: VR-1558) and HCoV-229E (ATCC: VR-740) were obtained from the ATCC. SARS-CoV-2 was propagated once in Vero/TMPRSS2 cells in DMEM-F12 containing 0.2% bovine serum albumin (BSA) at 37°C. HCoV-OC43 and HCoV-229E were propagated once in HCT8 cells and MRC5 cells respectively in DMEM-F12 containing 0.2% BSA at 33°C. All viral stocks used in this study were prepared by limiting dilutions of the provided original stocks to eliminate potential inclusion of defective interfering particles in the stocks. Virus titration was performed by measuring focus-forming units (FFU) in focus-forming assays^54^^,^^55^^,^^56^ or the median tissue culture infective dose (TCID_50_) method on Vero cells (SARS-CoV-2 and HCoV-OC43) and MRC5 cells (HCoV-229E).

#### Viral infection of cells in cultures

Human cells and Vero were infected with CoVs at a multiplicity of infection (MOI) of 0.001. After 1 h of incubation at 37°C, the cells were washed twice with phosphate-buffered saline (PBS), maintained in DMEM-F-12 medium containing 0.2% BSA, and incubated at 33°C or 37°C.

#### Immunostaining and confocal microscopy

Cells infected with CoVs were fixed at the indicated times post-infection with 4% paraformaldehyde in PBS for 15 min followed by permeabilization with 0.2% Triton-X for 20 min at room temperature. The infected cells were stained with rabbit antibodies against SARS-CoV-2 NP (GENETEX), HCoV-OC43 NP and HCoV-229E NP (Sino Biological) for 1 h at 37°C, followed by incubation with a secondary antibody conjugated with Alexa Fluor-488 (Invitrogen) at 37°C for 1 h. To confirm the IVT RNA secondary structures, 293T cells were transfected with IVT RNAs and fixed 24 h later, followed by permeabilization as described above. The cells were stained with mouse anti-dsRNA J2 antibody (SCICONS) for 1 h at 37°C, followed by incubation with Alexa Fluor-488 secondary antibody at 37°C for 1 h. Hoechst 33342 (Invitrogen) was used for the counterstaining of nuclei. Immunofluorescence images were captured using an FV3000 confocal laser scanning microscope (OLYMPUS). Foci for dsRNA were quantified using cellSens imaging software (OLYMPUS) from eight randomly selected image fields.

#### ELISA

The amounts of IFN-β and IL-6 in cell-culture supernatants were quantified using Quantikine kits (R&D Systems), according to the manufacturer’s instructions. The amounts of IFN-β and IL-6 in hamster lung homogenates were quantified using Nori Hamster ELISA kits (Genorise Scientific), according to the manufacturer’s instructions. Optical density at 450 nm was measured with an SH-9000 lab microplate reader (Corona Electric).

#### Immunoblotting

Cell lysis and immunoblot analysis were performed as described previously.^56^^,^^57^ Briefly, the samples were resolved by SDS-PAGE and transferred onto polyvinylidene difluoride membranes (Millipore). Western blotting was performed and the bands were visualized with the Amersham ECL Select Western blotting detection reagent and an Amersham Imager 680 (GE Healthcare). The band intensities were quantified by Amersham Imager 680 Analysis software (GE Healthcare).

#### Total RNA transfection

Total RNAs were extracted from Vero cells mock-infected or infected with SARS-CoV-2 using miRNeasy Mini kits (QIAGEN), fractionated into small (<200-nt) RNAs and large (>200-nt) RNAs according to the manufacturer’s instructions, and transfected at a final concentration of 0.6 μg/mL or 6 μg/mL into 293T cells and THP-1 cells in 24-well culture plates using Transit-mRNA (Mirus). Proper fractionation of small and large RNAs was verified by the 2100 Bioanalyzer system (Agilent) before use. Poly (I:C) was included as a positive control. At 24 h post-transfection, cell culture supernatants were collected to measure IFN-β and IL-6 levels by ELISA as described above.

#### RNA fractionation from CoV-infected cells and enzyme treatment for small RNA sequencing

Total RNAs from CoV-infected cells were isolated shortly before the times when titers plateaued at which times cytopathic effects were yet not apparent (72 hpi for CoVs in human/Vero cells at 33°C except 48 hpi for SARS-CoV-2 in Vero cells and at 37°C, 48 hpi and 24 hpi for SARS-CoV-2 in Calu-3 cells and Vero cells, respectively). Total RNAs were extracted and fractionated into small and large RNAs as described above and the small RNA fraction was then treated as described previously^27^ with some modifications. Briefly, the small RNA fraction was treated with XRN-1 (New England BioLabs) in NEB buffer 2 specific for converting 5′PPP to 5′ P and incubated at 37°C for 1 h to digest host-derived miRNAs harboring 5′ P. XRN-1 was inactivated by incubating at 70°C for 10 min. 5′ PPP RNAs derived from viral RNAs were converted to monophosphorylated RNAs by RppH treatment at 37°C for 1 h. The enzyme-treated small RNAs were purified using RNA Clean & Concentrator™-25 (ZYMO RESEARCH). The prepared RNAs were applied to RNA-sequencing as described below.

#### Small RNA sequencing

A miRNA library was constructed using the NEBNext Multiplex Small RNA Library Prep Set for Illumina (NEB) following the manufacturer’s instructions. For this, 0.05 ng of RNA was reversed transcribed into cDNA after ligation of the multiplex 3′ SR Adaptor, hybridization of the reverse transcription primer, and ligation of the multiplex 5′ SR Adaptor. The RNA library was then amplified by 20 PCR cycles using Illumina compatible index primers. The amplified library was resolved on a 2% E-Gel EX agarose gel (Thermo Fisher). DNA fragments corresponding to approximately 150-350 bp (small RNA inserts plus 3′ and 5′ adaptors) were recovered using QIAquick Gel Extraction Kits (QIAGEN). The library was quantified by Qubit fluorometer (Thermo Fisher) and sequenced on the Illumina NovaSeq 6000 platform using paired end reads (100 bp). The sequencing generated >1,000,000 raw reads from the sample. Adaptor sequences were removed from the raw sequencing reads using the Cutadapt program. The trimmed reads were mapped to the SARS-CoV-2 parental strain genome (LC522975) or the Delta strain (EPI_ISL_2158617) using HISAT2 version 2.1.0 (options: --pen-noncansplice 0 --no-temp-splicesite --no-softclip --pen-canintronlen G,0,0 --pen-noncanintronlen G,0,0). The trimmed reads were also mapped to the host genome (human reference genome sequence (hg19) or *Chlorocebus sabaeus* strain WHO RCB 10-87 unplaced genomic scaffold, Vero_WHO_p1.0 scaffold-1, whole genome shotgun sequence (NW_023666033)) using HISAT2 version 2.1.0 (option: --no-softclip).

#### Large RNA-sequencing

Full-length cDNA was generated using a SMART-Seq HT Kit (Takara Bio) according to the manufacturer’s instructions. An Illumina library was prepared using a Nextera DNA Library Preparation Kit (Illumina, San Diego, CA) according to the SMARTer kit instructions. Sequencing was performed using an Illumina NovaSeq 6000 sequencer (Illumina) in the 100-base paired-end mode. Illumina RTA3 v3.4.4 software was used for base calling. Primer sequences were removed from the raw sequencing reads using the Cutadapt program. The trimmed reads were mapped to the SARS-CoV-2 genome of the parental strain (LC522975) or the Delta strain (EPI_ISL_2158617) using HISAT2 version 2.1.0 (options: --pen-noncansplice 0 --no-temp-splicesite --no-softclip --pen-canintronlen G,0,0 --pen-noncanintronlen G,0,0).

#### *In vitro* production of RNA fragments

DNA fragments corresponding to the first 40, 60, 80, and 100-nt in the 5′ UTR of SARS-CoV-2, HCoV-OC43 and HCoV-229E genomes were amplified by PCR using specific forward primers containing the T7 promoter sequence (ATTGTAATACGACTCACTATAGGG) and a *Hind III* restriction site, and specific reverse primers containing an *XbaI* restriction site. Amplified fragments were digested with *HindIII* and *XbaI* and cloned into enzyme-digested pUC18. Plasmid DNAs were linearized with *XbaI*, purified with QIAquick PCR Purification Kits (QIAGEN) and used as templates for T7 *in vitro*-transcription using the T7 RiboMAX™ Express Large Scale RNA Production System (PROMEGA). IVT RNAs were HPLC-purified and then further purified with miRNeasy mini kits. For preparation of dephosphorylated RNAs, IVT RNAs were treated with CIP for 1 h at 37°C and the treated RNAs were then purified with RNA Clean & Concentrator™-25 (ZYMO RESEARCH). Ribo m7G Cap Analog (PROMEGA) was used to synthesize Cap-0 RNA transcripts. Cap-0 RNAs were then converted to Cap-1 RNAs with mRNA Cap 2′-O-Methyltransferase (NEB) and purified with RNA Clean & Concentrator™-25. IVT RNAs were transfected into 293T cells and PMA-stimulated THP-1 cells using Transit-mRNA (Mirus). At 24 h post-transfection, cell culture supernatants were collected to measure IFN-β and IL-6 levels by ELISA as described above.

#### siRNA transfection

siRNA for RIG-I (GACUAGUAAUGCUGGUGUAUU with dTdT overhangs) was chemically synthesized by a gene synthesis service (Fasmac). Silencer™ Select Pre-Designed siRNA was used for MDA5 silencing (s34499; Thermo Fisher). siRNAs were transfected using Transit-TKO (Mirus) and incubated for 48 h. To validate silencing, total RNAs were isolated from siRNA-transfected cells using RNeasy Mini Kits (QIAGEN) and qPCR was performed for RIG-I (Hs01061436_m1; Thermo Fisher), MDA5 (Hs00223420_m1; Thermo Fisher) and 18s ribosomal RNA (Hs99999901_S1; Thermo Fisher). Data were normalized to the expression levels of 18S ribosomal RNA for each sample and the ΔΔCt method was used for the relative value quantification.

#### IRF luciferase assay

A final concentration of 2 μg/mL small (<200-nt) RNA fractions or 10^10^ copies of IVT RNAs prepared as described above were transfected into an equal number of THP-1-dual (IRF reporter) cells, THP-1-dual KO-MDA5 cells or THP-1-dual KO-RIG-I cells in 96-well plates using Transit-mRNA (Mirus). At 24 h post-transfection, cell culture supernatants were collected to monitor IRF/type 1 IFN signaling by measuring ISRE-driven Lucia luciferase activity with QUANTI-Luc reagents (Invivogen). Light signals produced were measured with an SH-9000lab microplate reader (Corona Electric) and used for relative value quantification.

#### Stem-loop qRT-PCR to quantify 5′ end svRNA levels

Total RNA was extracted from virus-infected cells at the indicated time points, or from lungs and sera of hamsters 5 dpi using miRNeasy mini kits. RNA was reverse transcribed using TaqMan MicroRNA Reverse Transcription Kits (Applied Biosystems) and qPCR was performed using TaqMan Fast Advanced Master Mix (Applied Biosystems) according to the manufacturer’s instructions. Stem-loop RT primer, primers and probes for qPCR were designed by Custom TaqMan Small RNA Assays (Applied Biosystems) based on the representative sequences of 5′ end svRNA from SARS-CoV-2 (ATTAAAGGTTTATACCTTCCCAGGTAACAAACCAACCAACTTTCGATCTCTTGTAGATCTGTT) and HCoV-OC43 (ATTGTGAGCGATTTGCGTGCGTGCATCCCGTTTCACTGATCTCTTGTTAGATCTTTTTGTAATCTAAACTTT). The specificity of the SARS-CoV-2 63-nt specific stem-loop qRT-PCR was tested using corresponding 5′ end svRNA templates with different 5′ end lengths (43, 63, 150 and 265-nt) that were transcribed *in vitro*, followed by HPLC-purification as described above. The specificity of the HCoV-OC43 72-nt specific stem-loop qRT-PCR was also tested using corresponding 5′ end svRNA templates with different 5′ end lengths (42, 72, 140 and 265-nt) that were *in vitro* transcribed in the same manner. For Calu-3 cell samples, TaqMan MicroRNA Assays for snRNA-U6 (001973) were used as controls for normalization of small RNAs from cells and the relative expression was calculated by the ΔΔCt method using the 8 hpi samples as a reference with a set value of one relative unit. For hamster samples, 100 ng total RNA were used for qRT-PCR.

#### qRT-PCR to quantify SARS-CoV-2 N mRNA levels

Total RNA was extracted from virus-infected cells at the indicated time points using miRNeasy mini kits. qRT-PCR was performed using QuantiTect Probe RT-PCR Kits (Applied Biosystems) according to the manufacturer’s instructions and a primer-probe set specific for SARS-CoV-2 N mRNA (N1 set) designed by the Japan National Institute of Infectious Disease (NIID): N_Sarbeco_F1 (CACATTGGCACCCGCAATC) and N_Sarbeco_R2 (GAGGAACGAGAAGAGGCTTG) and FAM-TAMRA-labeled specific probe N_Sarbeco_P1 (FAM-ACTTCCTCAAGGAACAACATTGCCA-TAMRA). This primer-probe set targets the central region of N mRNA (nucleotide residues 28723-28850) that does not overlap the leader region covered by the stem-loop RT primer-probe set.

#### Virus infection of *ex vivo* reconstituted human nasal epithelia

The MucilAir system is a reconstituted human nasal epithelium, consisting of ciliated, goblet and basal cells. Cultures were maintained under air/liquid interface (ALI) conditions in transwells with 700 μL MucilAir medium in the basal compartment. Prior to viral infection, the apical surface was washed twice with 200 μL MucilAir medium (20 min at 37°C) to remove mucus. Cells were infected with SARS-CoV-2 or HCoV-C43 on the apical side at an MOI of 0.1 in 150 μL MucilAir medium for 1.5 h at 37°C. Viral inoculum was removed and cells were washed twice with MucilAir medium (20 min at 37°C) before continuing culture for 24, 48 and 96 h. Apical supernatants were harvested by adding 200 μL MucilAir medium on the apical side and incubating for 20 min at 37°C prior to collection, after which viral titers were determined by FFU assays. Intracellular RNA of each well was harvested using miRNeasy mini kits and 5′ end svRNA levels were quantified by stem-loop qRT-PCR as described above. For SARS-CoV-2, a stem-loop primer specific for the most common 63-nt 5′ end svRNA was used, whereas for HCov-OC43, a stem-loop primer specific for the most common 72-nt 5′ end svRNA was used. Basal medium was harvested to measure IFN-β and IL-6 production levels as described above.

#### RNA immunoprecipitation (RIP) assay

The RIP assay was conducted using the kit for microRNA (MBL) according to the manufacturer’s protocol. Calu-3 cells infected with SARS-CoV-2 at 72 hpi were lysed with lysis buffer and pre-cleared with Protein G Dynabeads (Thermo Fisher). The supernatants were incubated with Protein G immobilized with anti-RIG-I antibody (#3743; Cell Signaling) with gentle rotation for 3 h at 4°C. The beads were then washed ×3 with wash buffer. For RNA-seq, the sRNA fraction was extracted from the total RNA bound to the beads using RNeasy Mini Kits (QIAGEN).

#### IFN-β antagonism assay

A pcXN2 plasmid expressing SARS-CoV-2 ORF6 fused with a Flag-tag at the 3′ terminus (0, 50, 100, 150 or 300 ng per well) was transfected into 293T cells (in a 24-well plate) with Transit-LT1 Reagent (Mirus). At 24 h post-transfection, cells were stimulated with 2.5 × 10^11^ copies of IVT-RNA corresponding to the first 60-nt of the 5′ end from the SARS-CoV-2 genome using Transit-mRNA (Mirus). At 48 h post transfection, the cells and culture supernatants were harvested for Western blotting and ELISA to determine ORF6 expression and IFN-β production.

#### svRNA complementation assay

Vero cells in 24-well culture plates were infected with SARS-CoV-2 at an MOI of 0.001. After 1 h adsorption, cells were washed and transfected with 0, 10^4^, 10^8^, 10^12^ copies of IVT RNA corresponding to the representative SARS-CoV-2 5′ UTR svRNA having 63-nt, as described above. Total RNAs were then collected from cells 36 h post-infection and quantified by qRT-PCR using stem-loop primer sets specific for the 63-nt svRNA or N mRNA as described above.

Total RNAs were extracted from Vero cells mock-infected or infected with SARS-CoV-2 using miRNeasy Mini kits (QIAGEN), fractionated into small (<200-nt) RNAs and large (>200-nt) RNAs according to the manufacturer’s instructions, and transfected at a final concentration of 0.6 μg/mL or 6 μg/mL into 293T cells and THP-1 cells in 24-well culture plates using Transit-mRNA (Mirus). Proper fractionation of small and large RNAs was verified by the 2100 Bioanalyzer system (Agilent) before use. Poly (I:C) was included as a positive control. At 24 h post-transfection, cell culture supernatants were collected to measure IFN-β and IL-6 levels by ELISA as described above.

#### Hamster infection model

Four-week-old female Syrian hamsters were purchased from SLC Japan. Under mixed anesthesia (medetomidine-butorphanol-midazolam), the animals were inoculated intranasally with 1.0×10^6^ plaque-forming units SARS-CoV-2 (in 60 μL) as described previously.^38^ On day 5 post-infection, all animals were euthanized and lungs were collected for viral titration and/or stem-loop qRT-PCR.

### Quantification and statistical analysis

Data analyses were performed using GraphPad Prism Version 6 software (GraphPad Software). Statistically significant differences between virus pairs were determined by ANOVA with Tukey’s multiple comparison test. Data are presented as the means ± SD.

## Data Availability

Data reported in the study are available from the lead contact upon request. This paper doses not report original code. Any additional information required to reanalyse the data reported in this paper is available from the lead contact upon request.
