# Supplementary material for: Stimulation of interferon-β responses by aberrant SARS-CoV-2 small viral RNAs acting as retinoic acid-inducible gene-I agonists
Source: iScience. 2022 Dec 7;26(1):105742. doi: 10.1016/j.isci.2022.105742 (PMC9726650; doi:10.1016/j.isci.2022.105742)
Supplement: Document S1. Figures S1–S4 and Tables S1–S6 [file mmc1.pdf]

iScience, Volume *26*

Supplemental information

Stimulation of interferon-β responses by aberrant SARS-CoV-2 small viral RNAs acting as retinoic acid-inducible gene-I agonists

Yasuha Arai, Itaru Yamanaka, Toru Okamoto, Ayana Isobe, Naomi Nakai, Naoko Kamimura, Tatsuya Suzuki, Tomo Daidoji, Takao Ono, Takaaki Nakaya, Kazuhiko Matsumoto, Daisuke Okuzaki, and Yohei Watanabe

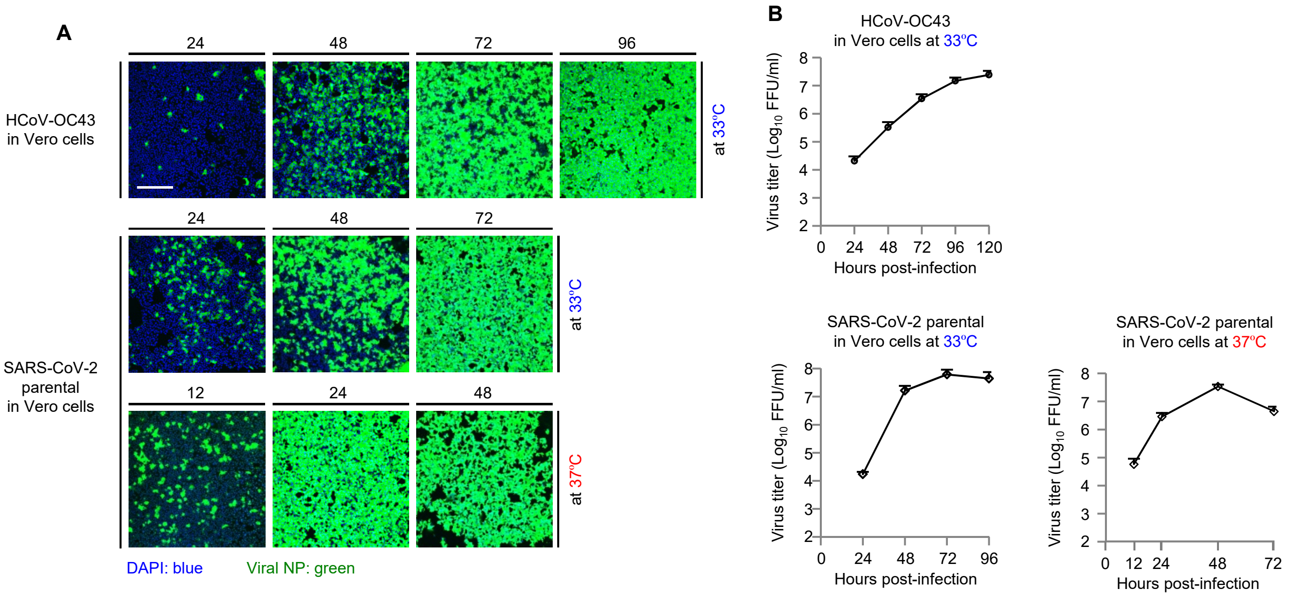

Figure S1. Replication kinetics of SARS-CoV-2 and HCoV-OC43 in Vero cells, Related to Figure 1.
Vero cells were infected with CoVs at an MOI of 0.001 and incubated at 33ºC (for HCoV-OC43) and both at 33ºC and 37ºC (for SARS-CoV-2). **(A)** Confocal microscopy images of CoV-infected Vero cells. At the indicated times post-infection, cells were stained with anti-viral NP antibodies (green). Nuclei were stained with Hoechst 33342 (blue). Scale bar = 250 μm. **(B)** The progeny virus titers were measured by FFU assays at the indicated times post-infection. Each data point is the mean ± SD from three independent experiments.

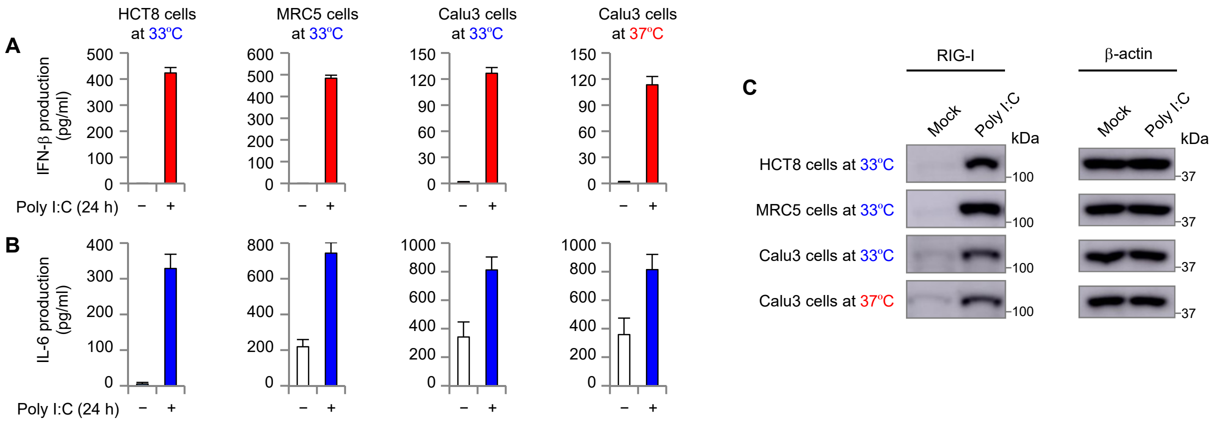

Figure S2. Substantial antiviral signaling pathways in human Calu-3, MRC5 and HCT8 cells promote rapid IFN-β and IL-6 production, Related to Figure 1.

(A and B) Induction of IFN-β and IL-6 secretion from human cells by poly (I:C) transfection. Cells cultured in 24-well plates were stimulated by poly (I:C) transfection. At 24 h after stimulation, the supernatants were harvested for ELISA. Each data point is the mean ± SD from three independent experiments. **(C)** Immunoblot analysis of RIG-I in the lysates of poly (I:C)-stimulated human cells at 24 h post-transfection. Representative images from two independent experiments are shown.

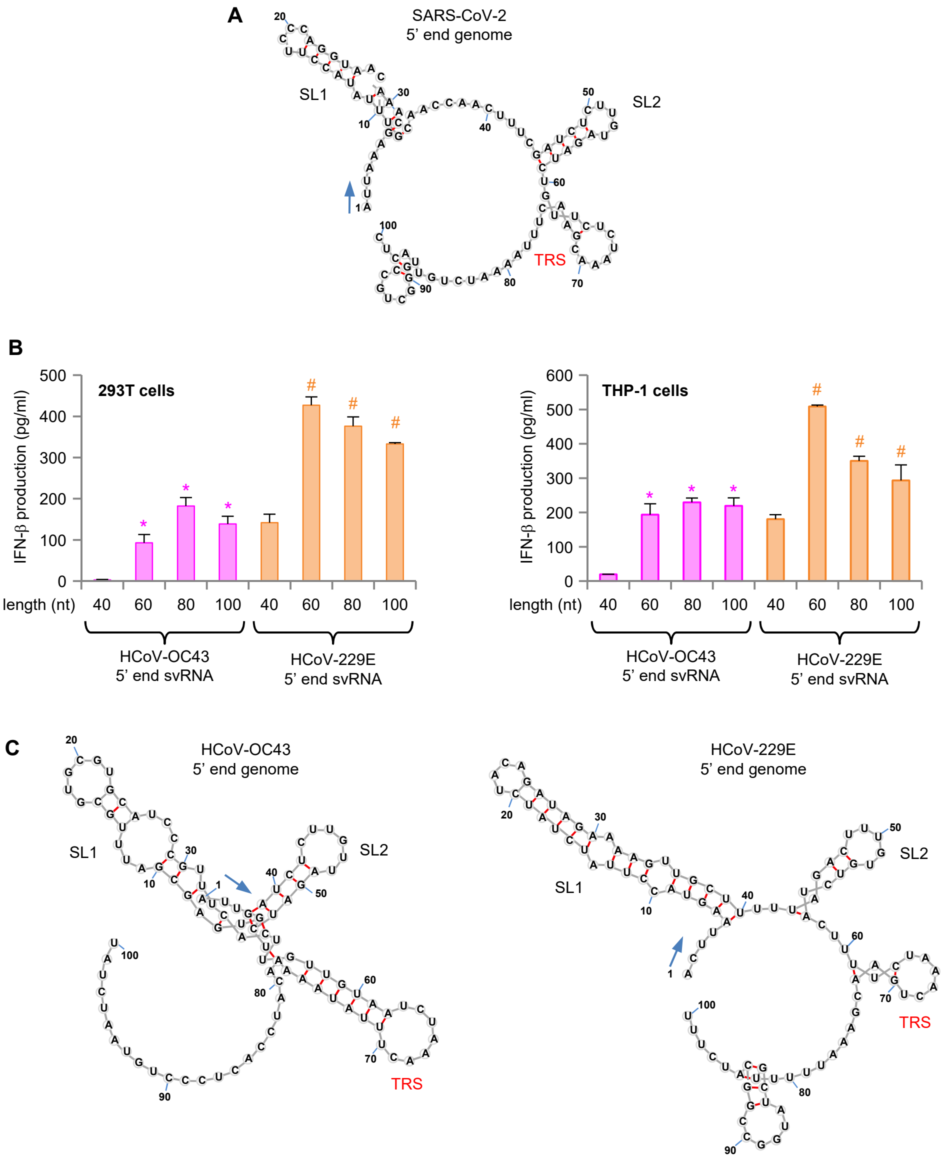

Figure S3. HCoV-OC43 and HCoV-229E 5′ end svRNAs have IFN-b stimulatory ability, Related to Figure 5.
(A) Secondary structure of 100-nt IVT 5′ UTR svRNA of SARS-CoV-2, predicted by RNAfold with default parameters. SL indicates stem-loop. **(B)** Induction of IFN-β secretion from 293T cells and differentiated THP-1 cells by transfection of IVT 5′ UTR svRNAs from HCoV-OC43 and HCoV-229E. Cells cultured in 24-well plates were stimulated by transfection of 40, 60, 80 and 100-nt IVT 5′ UTR svRNAs with the same amounts of RNA as in **Figure 5B**. At 24 h after stimulation, the supernatants were harvested for ELISA. Each data point is the mean ± SD from three independent experiments. Statistically significant differences compared to IVT 40-nt svRNAs from HCoV-OC43 (*P < 0.01) or from HCoV-229E (#P < 0.01) are shown. **(C)** Secondary structure of 100-nt IVT 5′ UTR svRNA of HCoV-OC43 (left) and HCoV-229E (right), predicted by RNAfold with default parameters. SL indicates stem-loop.

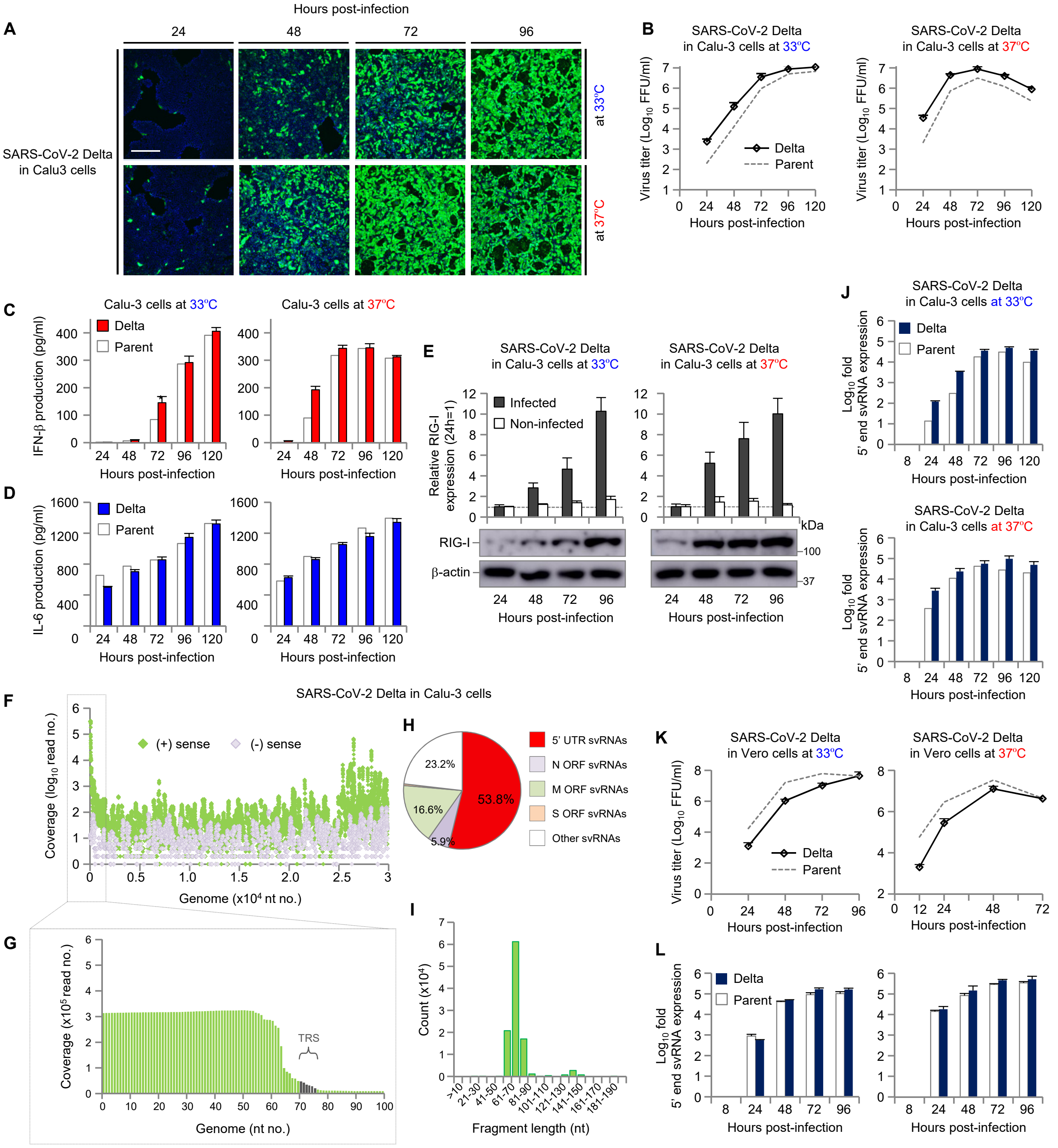

Figure S4. SARS-CoV-2 Delta produces 5′ end svRNAs and leads to IFN induction similar to the parental virus, Related to Figures 1-4.
(A and B) Replication kinetics of the SARS-CoV-2 Delta in human Calu-3 cells. Cells were infected with Delta at MOIs of 0.001 and incubated at 33ºC or 37ºC. **(A)** Confocal microscopy images of Calu-3 cells infected with Delta. At the indicated times post-infection, cells were stained with anti-viral NP antibody (green). Nuclei were stained with Hoechst 33342 (blue). Scale bar = 250 µm. **(B)** The progeny virus titers were measured by FFU assays at the indicated times post-infection. **(C and D)** Induction of IFN-β **(C)** and IL-6 **(D)** secretion from Calu-3 cells infected with Delta. At the indicated times post-infection, the supernatants were harvested for ELISA. **(E)** Immunoblot analysis of RIG-I in lysates of the Delta-infected Calu-3 cells at the indicated times post-infection. Representative images of three independent experiments are shown. Quantification of band intensity is relative to 24 h post-infection. Each data point is the mean ± SD from three independent experiments. **(F)** sRNA-seq reads mapped to Delta genomes. Reads were strand-specifically mapped to the positive-sense (+) RNAs or negative-sense (-) RNAs. Read counts were quantified for each nucleotide of the genome. **(G)** Read map of 5′ UTR svRNAs produced in Delta-infected Calu-3 cells. Reads were mapped to the positive-sense RNA. Read counts were quantified for each nucleotide. **(H)** Fraction of svRNA fragments produced by Delta, according to the color legend on the figure. **(I)** Size distribution of 5′ UTR svRNAs. **(J)** RT-qPCR quantification of 5′ end svRNA levels at 8, 24, 48, 72, 96 and 120 hpi in Delta-infected Calu-3 cells at 33ºC or 37ºC as indicated in the legend to **Figure 1**. Levels of 5′ end svRNAs were related to those at 8 hpi, as calculated by the ΔΔCt method using snRNA-U6 as an endogenous control. Each data point is the mean ± SD of three independent experiments. **(K and L)** Vero cells were infected with the SARS-CoV-2 Delta variant **(F)** at 33ºC or 37ºC as indicated in the legend to **Figure 1**. **(K)** Replication kinetics of Delta in Vero cells. **(L)** RT-qPCR quantification of 5′ end svRNA levels at 8, 24, 48, 72 and 96 hpi in Vero cells infected with Delta. Levels of 5′ end svRNAs were related to those at 8 hpi, as calculated by the ΔΔCt method using snRNA-U6 as an endogenous control. Each data point is the mean ± SD from three independent experiments.

Table S1. Summary of sRNA libraries from CoV-infected cells, Related to Figures 2-4.

Analysis	Host	Sample	Total CoV-infected cell no.	Total no. of read	Host read		CoV read		% of CoV read
					No.	Relative[a]	No.	Relative[a]	
No. 1	Human	SARS-CoV-2 in Calu3 cells	41.7×10^5	28,707,198	24,685,521	1.34	444,421	27.33	1.80
		HCoV-229E in MRC5 cells	39.2×10^5	30,676,346	25,052,444	1.34	139,640	8.59	0.56
		HCoV-OC43 in HCT8 cells	42.7×10^5	21,651,320	18,459,950	1.0	16,261	1.0	0.09
No. 2	African Green Monkey	SARS-CoV-2 in Vero cells	27.8×10^5	34,236,924	30,228,339	1.06	743,719	4.48	2.46
		HCoV-OC43 in Vero cells	27.8×10^5	32,704,636	28,475,034	1.0	165,871	1.0	0.58

[a]Relative to read no. from HCoV-OC43-infected HCT8/Vero cells.

Table S2. Summary of lRNA libraries from CoV-infected cells, Related to Figures 2-4.

Analysis	Host	Sample	Total CoV-infected cell no.	Host read	CoV read	
					No.	Relative[a]
No. 1	Human	SARS-CoV-2 in Calu3 cells	41.7×10^5	17,924,773	23,692,418	9.21
		HCoV-229E in MRC5 cells	39.2×10^5	14,714,770	7,635,600	2.96
		HCoV-OC43 in HCT8 cells	42.7×10^5	16,340,177	2,571,861	1.0
No. 2	African Green Monkey	SARS-CoV-2 in Vero cells	27.8×10^5	13,383,483	17,619,106	2.67
		HCoV-OC43 in Vero cells	27.8×10^5	15,680,383	6,590,756	1.0

[a]Relative to read no. from HCoV-OC43-infected HCT8/Vero cells.

Table S3. Summary of categorized svRNA fragments from CoV-infected cells[a], Related to Figures 2-4.

Analysis	Host	Sample	5' UTR svRNAs		N/M/S ORF svRNAs		5' UTR svRNAs + N/M/S ORF svRNAs[b]	
			No.	Relative[c]	No.	Relative[c]	No.	Relative[c]
No. 1	Human	SARS-CoV-2 in Calu3 cells	122,574	44.0	37,242	21.7	159,816	35.5
		HCoV-229E in MRC5 cells	4,004	1.4	32,529	19.0	36,533	8.1
		HCoV-OC43 in HCT8 cells	2,784	1.0	1,715	1.0	4,499	1.0
No. 2	African Green Monkey	SARS-CoV-2 in Vero cells	208,874	9.6	39,036	1.9	247,910	5.9
		HCoV-OC43 in Vero cells	21,846	1.0	20,167	1.0	42,013	1.0

[a]numbers in Table S3 are related to Figures 3B and 3C.

[b]5' UTR svRNAs and N/M/S ORF svRNAs constitute most of the 5' end-containing svRNAs in CoV-derived svRNA species.

[c]Relative to fragment no. from HCoV-OC43-infected HCT8/Vero cells.

Table S4. Summary of sRNA library from SARS-CoV-2 Delta-infected cells, Related to Figures 2-4.

Sample	Total CoV-infected cell no.	Total no. of read	No. of host read	CoV read		% of CoV read
				No.	Relative[a]	
SARS-CoV-2 Delta in Calu-3 cells	41.7 x 10^5	64,572,126	51,943,529	406,766	0.92	0.63

[a]Relative to read no. from SARS-CoV-2 parental strain-infected Calu-2 cells.

Table S5. Summary of categorized svRNA fragments from SARS-CoV-2 Delta-infected cells, Related to Figures 2-4.

Sample	5' UTR svRNAs		N/M/S ORF svRNAs		5' UTR svRNAs + N/M/S ORF svRNAs	
	No.	Relative[a]	No.	Relative[a]	No.	Relative[a]
SARS-CoV-2 Delta in Calu-3 cells	105,093	0.86	45,301	0.61	150,044	0.77

[a]Relative to svRNA fragment no. from SARS-CoV-2 parental strain-infected Calu-3 cells.

Table S6. Mutations of replication-associated viral proteins in the SARS-CoV-2 strains used in this study, Related to Figure 7.

Viral protein	Position	Amino acid type in:			
		Parental	Delta	Omicron BA.1	Omicron BA.2
NSP12 (RDRP[a])	323	P	L	L	L
	671	G	S	G	G
NSP7 (RDRP[a] cofactor)	-[f]	-	-	-	-
NSP8 (RDRP[a] cofactor)	-	-	-	-	-
NSP10 (2'-O-MTase[b] cofactor)	-	-	-	-	-
NSP13 (RNA helicase)	77	P	L	P	P
NSP14 (exoRNase[c]/N7-MTase[d])	42	I	I	V	V
	394	A	V	A	A
NSP15 (endRNase[e])	112	A	V	A	A
NSP16 (2'-O-MTase[b])	-	-	-	-	-

[a]RDRP indicates RNA-dependent RNA polymerase.

[b]2'-O-MTase indicates 2'-O-methyltransferase.

[c]exoNNase indicates exoribonuclease.

[d]N7-MTase indicates guanine-N7-methyltransferase.

[e]endRNase indicates endoribonuclease.

[f]- indicates no amino acid mutations detected among the four virus strains used in this study.
